# Long-term exposure to ambient air pollution and lipid profiles: a longitudinal study in a heavily industrialized region of northern China

**DOI:** 10.3389/fpubh.2026.1950282

**Published:** 2026-10-06

**Authors:** Caiyun Sun, Xiaofeng Li, Yuanjia Miao, Jiayu Shen, Yang Jin, Feng Ma, Shigong Wang, Jingxin Li, Changxin Li

**Affiliations:** 1 First Clinical Medical College of Shanxi Medical University, Taiyuan, China; 2 Department of Neurology, The First Hospital of Shanxi Medical University, Taiyuan, China; 3 Yulin Meteorological Bureau, Yulin, Shaanxi, China; 4 Institute of Environmental Meteorology and Health, College of Atmospheric Sciences, Chengdu University of Information Technology, Chengdu, China; 5 State Key Laboratory of Severe Weather Meteorological Science and Technology, Institute of Tibetan Plateau Meteorology, Chinese Academy of Meteorological Sciences, Beijing, China

**Keywords:** ambient air pollution, ChinaHighAirPollutants (CHAP), dyslipidemia, lipid profiles, long-term exposure, repeated-measures study

## Abstract

**Background:**

Dyslipidemia is an important modifiable risk factor for cardiovascular disease, and growing evidence suggests that long-term ambient air pollution exposure may adversely affect lipid metabolism. However, longitudinal evidence from heavily industrialized regions with complex multipollutant exposure remains limited.

**Methods:**

We conducted a repeated-measures study of 9,040 adults with 19,113 lipid measurements from a health-screening program in Shanxi Province, China, between October 2019 and September 2023. Three-year average exposures to PM_2.5_, PM_10_, O_3_, SO_2_, NO_2_, and CO were estimated using the high-resolution ChinaHighAirPollutants (CHAP) dataset based on geocoded residential addresses. Linear mixed-effects models and generalized linear mixed-effects models were used to evaluate associations with lipid profiles and dyslipidemia, respectively. Restricted cubic spline, stratified, sensitivity, and quantile g-computation-style mixed-effects analyses were further conducted.

**Results:**

Higher long-term exposure to PM_2.5_, PM_10_, O_3_, NO_2_, and SO_2_ was associated with higher triglyceride (TG), total cholesterol (TC), and low-density lipoprotein cholesterol (LDL-C) levels and lower high-density lipoprotein cholesterol (HDL-C) levels, whereas CO showed weaker associations. Among pollutants, PM_2.5_ showed relatively pronounced associations; each 10 μg/m^3^ increase was associated with 1.85, 1.70, and 1.81% increases in TG, TC, and LDL-C, respectively, and a 1.55% decrease in HDL-C. Similar patterns were observed for dyslipidemia. Stratified analyses suggested potential effect modification by sex, age, and BMI for selected pollutant-lipid associations, with the most robust interaction signals observed for age-stratified HDL-C associations after false discovery rate (FDR) correction. The associations remained consistent in sensitivity analyses. Restricted cubic spline analyses suggested nonlinear exposure-response relationships between PM_2.5_ and lipid parameters, while the quantile g-computation-style mixed-effects analysis indicated that multipollutant exposure was associated with adverse lipid profiles, with PM_2.5_ and O_3_ showing relatively large weights in the overall mixed-exposure association.

**Conclusion:**

Long-term ambient air pollution exposure was associated with adverse lipid profiles and may be related to higher odds of dyslipidemia among adults in a heavily industrialized region of northern China. These findings suggest that ambient air pollution may be an important environmental factor associated with lipid metabolism and highlight the need to consider long-term multipollutant exposure in cardiometabolic health research and prevention strategies.

## Introduction

Dyslipidemia is a major modifiable risk factor for cardiovascular disease (CVD) and other cardiometabolic disorders. Its prevalence has increased substantially worldwide over the past two decades. In China, the prevalence of dyslipidemia among adults increased from 18.6% in 2002 to 35.6% in 2018, representing a growing public health challenge (1–3). Increasing evidence suggests that ambient air pollution may be associated with cardiometabolic health. Experimental studies have demonstrated that both particulate and gaseous pollutants may disrupt lipid metabolism through multiple biological pathways, including oxidative stress, systemic inflammation, endothelial dysfunction, and metabolic disturbances, thereby contributing to the development of atherosclerosis and cardiovascular disease (4–6).

Although epidemiological studies have reported associations between long-term exposure to ambient air pollution and adverse lipid profiles, findings remain inconsistent across different populations and regions (7, 8). Most existing studies have been conducted in developed countries or economically developed regions of China, while evidence from heavily industrialized areas with relatively high pollution levels remains limited. Moreover, previous studies have primarily evaluated the independent effects of single pollutants using conventional regression models, whereas potential nonlinear exposure-response relationships and the joint associations of multiple pollutants have received considerably less attention.

Shanxi Province is one of China’s largest coal-producing and energy-intensive regions and has long experienced relatively high levels of ambient air pollution due to intensive industrial activities and fossil fuel combustion (9). Although air quality has improved in recent years following the implementation of national clean air policies, complex multipollutant pollution remains an important environmental challenge (10, 11). Nevertheless, longitudinal epidemiological evidence regarding the associations between long-term air pollution exposure and lipid profiles in this region remains scarce.

Therefore, this study aimed to investigate the associations of long-term exposure to multiple ambient air pollutants with lipid profiles and dyslipidemia among adults in Shanxi Province using repeated health examination data. In addition, we examined potential effect modification, nonlinear exposure-response relationships, and the joint associations of multiple pollutants to better characterize the complex relationships between long-term air pollution exposure and lipid-related outcomes.

## Materials and methods

### Study population

This study was based on repeated health screening data from adults participating in the Stroke Risk Population Screening and Intervention Program in Shanxi Province, China. Data were obtained from the National Cerebrovascular Disease Big Data Platform^1^, which follows standardized data collection protocols and integrates nationwide stroke screening and follow-up records. Information on demographic characteristics, lifestyle factors, medical history, physical examinations, laboratory tests, and carotid ultrasound findings was collected using standardized questionnaires and examination protocols from the Stroke Risk Population Screening and Intervention Program.

A total of 10,219 participants who underwent stroke risk screening in Shanxi Province between October 2019 and September 2023 were initially considered for inclusion. Exclusion criteria were applied sequentially to avoid double-counting participants who met more than one criterion. Participants were excluded if they (1) had incomplete residential address information or had lived at their current residence for less than five consecutive years (*n* = 348); (2) had fewer than two valid lipid profile measurements during follow-up (*n* = 516); and (3) had missing data for key study variables, including lipid measurements, exposure assessment variables, or major covariates (*n* = 315). After applying these criteria, 9,040 participants with 19,113 repeated lipid measurements were included in the final analysis. The participant selection process is shown in Supplementary Figure S1. This study was approved by the Ethics Committee of the First Hospital of Shanxi Medical University (Approval No. KYLL-2024-182).

### Exposure assessment

Air pollutant data were obtained from the ChinaHighAirPollutants (CHAP) dataset, a series of high-resolution, full-coverage gridded products of ground-level ambient air pollutant concentrations across China. CHAP products integrate ground monitoring observations, satellite remote-sensing products, atmospheric reanalysis, land-use information, emission-related predictors, and other geospatial variables using machine-learning-based spatiotemporal modeling approaches (12–15). In this study, annual CHAP products for PM_2.5_, PM_10_, O_3_, NO_2_, SO_2_, and CO were used. Concentrations of PM_2.5_, PM_10_, O_3_, NO_2_, and SO_2_ were expressed in μg/m^3^, whereas CO was expressed in mg/m^3^. The O_3_ metric was defined as ground-level maximum daily 8-h average ozone. Detailed information on the CHAP product versions, temporal coverage, spatial resolution, pollutant-specific metrics, validation performance, and corresponding references is provided in Supplementary Table S3. Each participant’s stable residential address was geocoded into longitude and latitude. Participants with incomplete residential address information or residence duration of less than five consecutive years at the current residence were excluded. For each lipid examination, pollutant concentrations were assigned according to the CHAP grid cell corresponding to the participant’s geocoded residential coordinates. Among the 9,040 included participants, all 19,113 lipid examination records were successfully linked to CHAP exposure estimates. Long-term exposure was defined as the average pollutant concentration during the three complete calendar years before the year of lipid examination.

Meteorological data were obtained from the Global Surface Summary of the Day (GSOD) dataset maintained by the National Centers for Environmental Information of the National Oceanic and Atmospheric Administration. For each lipid examination record, the participant’s geocoded residential coordinates were matched to the nearest weather station with available meteorological data during the corresponding exposure window. Temperature and dew point temperature were converted from Fahrenheit to degrees Celsius. Relative humidity was calculated from temperature and dew point temperature using the standard Magnus formula and constrained to the physically plausible range of 0–100%. Three-year average temperature and relative humidity were calculated using the same exposure window as the air pollutants.

### Lipid measurements and dyslipidemia definition

Lipid measurement data were obtained from the National Cerebrovascular Disease Big Data Platform. However, examination-specific information on lipid-lowering medication use, including medication status, drug type, and treatment timing, was not available from this platform; therefore, lipid-lowering medication use could not be incorporated into the statistical models. Information on hypertension, coronary heart disease, and diabetes was available and was additionally adjusted for in extended sensitivity models, but these variables could not serve as reliable proxies for lipid-lowering medication use. According to the standardized technical protocol of the Stroke Risk Population Screening and Intervention Program, participants fasted for at least 8 h before peripheral venous blood collection. Serum total cholesterol (TC), triglycerides (TG), low-density lipoprotein cholesterol (LDL-C), and high-density lipoprotein cholesterol (HDL-C) were measured as part of the routine laboratory assessment using program-specified clinical laboratory procedures. After field collection, data were uploaded to the platform and underwent standardized quality-control checks, including completeness and plausibility checks by field investigators and screening institutions. Dyslipidemia was defined according to the Chinese Guidelines for the Prevention and Treatment of Dyslipidemia in Adults (2007). Hypercholesterolemia was defined as TC ≥ 6.22 mmol/L, hypertriglyceridemia as TG ≥ 2.26 mmol/L, low HDL-C as HDL-C < 1.04 mmol/L, and high LDL-C as LDL-C ≥ 4.14 mmol/L. Participants with one or more abnormal lipid parameters were classified as having dyslipidemia.

### Covariates

Covariates were selected *a priori* based on previous epidemiological studies of air pollution and lipid profiles to reduce potential confounding. We considered demographic and socioeconomic factors, lifestyle factors, anthropometric status, clinical history, and meteorological conditions as potential confounders of the associations between long-term air pollution exposure and lipid outcomes. Demographic and socioeconomic covariates included age, sex, marital status, educational level, and annual personal income. Lifestyle covariates included smoking status, alcohol consumption, and physical activity. Smoking status, alcohol consumption, and physical activity were self-reported using standardized questionnaires from the screening program and categorized according to the original questionnaire responses available in the National Cerebrovascular Disease Big Data Platform. Smoking status was classified as current smoking or non-smoking, alcohol consumption as current drinking or non-drinking, and regular physical activity was defined as physical activity at least three times per week and at least 30 min per session. Anthropometric status was assessed using body mass index (BMI). Clinical history included hypertension, coronary heart disease, and diabetes.

Covariates were handled according to their measurement structure in the repeated-measures dataset. Sex and educational level were treated as time-invariant covariates. Other covariates were matched to the corresponding lipid examination record and included as visit-specific covariates in the repeated-measures models. Meteorological covariates, including three-year average temperature and relative humidity, were included in the adjusted models to account for potential confounding by weather conditions.

### Statistical analysis

Continuous variables are presented as means ± standard deviations (SD) or medians with interquartile ranges (IQR), as appropriate, whereas categorical variables are summarized as frequencies and percentages. Pairwise correlations among PM_2.5_, PM_10_, O_3_, NO_2_, SO_2_, and CO were assessed using Spearman correlation coefficients. For multiple interaction tests, nominal *p*-values were additionally adjusted using the Benjamini–Hochberg false discovery rate (FDR) procedure.

For lipid levels, linear mixed-effects models (LMMs) were used to estimate the associations between long-term air pollutant exposure and natural-log-transformed TG, TC, LDL-C, and HDL-C levels. For dyslipidemia outcomes, generalized linear mixed-effects models (GLMMs) were used to estimate odds ratios and 95% confidence intervals. All mixed-effects models included participant as a random intercept to account for within-participant correlation across repeated lipid measurements, and all covariates were included as fixed effects. We also conducted a secondary incident-outcome analysis to evaluate whether the findings were consistent after excluding participants with dyslipidemia at baseline. For lipid-specific outcomes, baseline cases of the corresponding abnormal lipid parameter were excluded; for overall dyslipidemia, participants with any abnormal lipid parameter at baseline were excluded. Incident dyslipidemia was defined as the first detection of the corresponding abnormal lipid parameter during follow-up. Each eligible participant contributed one record to the analysis. The models used baseline 3-year average air pollutant exposure and were adjusted for the same covariates as the primary analyses, with additional adjustment for baseline lipid level, follow-up time, lipid examination year, and season. Pollutant concentrations were scaled before modeling, corresponding to 10 μg/m^3^ increments for PM_2.5_, PM_10_, O_3_, SO_2_, and NO_2_, and a 0.1 mg/m^3^ increment for CO. Accordingly, percent changes in lipid levels were calculated as [exp(β) – 1] × 100%, where β represents the regression coefficient for the corresponding pollutant increment. All mixed-effects models were fitted using the lme4 package in R. Model convergence and singular fit were checked for the main analyses. For linear mixed-effects models of continuous lipid outcomes, residual-based diagnostics were assessed using residual-versus-fitted plots and Q–Q plots; representative diagnostic plots are provided in Supplementary Figure S3.

Potential effect modification was evaluated by sex (male/female), age (<60/≥60 years), and BMI (<24/≥24 kg/m^2^). Stratified analyses were performed for each subgroup, and multiplicative interaction terms between pollutant concentrations and subgroup variables were incorporated into the models to test statistical interaction. To evaluate the robustness of the findings, several sensitivity analyses were performed. First, we repeated the main analyses using alternative exposure windows based on 1-year and 5-year average pollutant concentrations before each lipid examination. Second, we fitted an extended model with additional adjustment for histories of hypertension, diabetes, and coronary heart disease. Third, to assess the potential influence of temporal variation and regional spatial structure, we further adjusted for calendar year, season of examination, and prefecture-level region. Finally, for dyslipidemia outcomes, we repeated the analyses using definitions based on the updated 2023 Chinese lipid-management cutoffs. To provide additional insight into the associations between long-term air pollution exposure and lipid outcomes, restricted cubic spline (RCS) analyses were performed using natural cubic spline terms with 3 degrees of freedom in linear mixed-effects models to examine potential nonlinear exposure-response relationships between long-term PM_2.5_ exposure and lipid levels. The median PM_2.5_ concentration was used as the reference value, and *p*-values for overall association and nonlinearity were obtained using likelihood ratio tests. We further conducted a quantile g-computation-style mixed-effects analysis to evaluate the joint association of long-term exposure to the air pollutant mixture with lipid levels. Each pollutant was transformed into quartile scores, and all pollutant scores were entered simultaneously into mixed-effects models for lipid outcomes, with participant included as a random intercept. The overall mixture effect was interpreted as the percent change in lipid levels associated with a simultaneous one-quartile increase in all pollutants. Signed pollutant weights were calculated from pollutant-specific coefficients to describe the relative weights of individual pollutants in the overall mixed-exposure association. Because PM_2.5_ is a component of PM_10_, we additionally performed sensitivity mixture analyses excluding PM_10_ and replacing PM_10_ with the coarse particle fraction, defined as PM_10_ minus PM_2.5_.

All statistical analyses were performed using R software (version 4.4.1). All statistical tests were two-sided, and nominal *p*-values < 0.05 were considered statistically significant.

## Results

### Characteristics of the study population

A total of 9,040 participants were included in this repeated-measures study. Baseline characteristics are shown in Table 1 based on the first lipid examination. The mean age of the participants was 58.57 ± 10.81 years, 54.3% were women, 92.9% were married, and 61.3% had a BMI ≥ 24 kg/m^2^.

**Table 1:** Characteristics of study participants at the first lipid examination (*n* = 9,040).

Participants	9,040
Age (years), mean ± SD	58.57 ± 10.81
Sex, *n* (%)	
Female	4,907 (54.3)
Male	4,133 (45.7)
Marital status, *n* (%)	
Married	8,402 (92.9)
Widowed/Single/Divorced	638 (7.1)
Education level, *n* (%)	
Primary school or below	1,678 (18.6)
Junior high school or senior high school	6,244 (69.1)
University or above	1,118 (12.4)
Annual personal income (Yuan/year), *n* (%)	
<5,000	2064 (22.8)
5,000–10,000	1,396 (15.4)
10,000–20,000	1,199 (13.3)
≥20,000	4,381 (48.5)
Smoking, *n* (%)	
No	7,633 (84.4)
Yes	1,407 (15.6)
Drinking, *n* (%)	
No	7,278 (80.5)
Yes	1762 (19.5)
Physical activity, *n* (%)	
Regular	4,821 (53.3)
Irregular	4,219 (46.7)
BMI, *n* (%)	
<24	3,503 (38.8)
≥24	5,537 (61.3)
Temperature (°C), mean ± SD	12.50 ± 0.53
Relative humidity (%), mean ± SD	50.33 ± 2.36
Lipid profiles and dyslipidemia	
TG (mmol/L), median (P_25_, P_75_)	2.05 (1.87, 2.23)
TC (mmol/L), median (P_25_, P_75_)	5.63 (5.34, 5.95)
LDL-C (mmol/L), median (P_25_, P_75_)	3.62 (3.37, 3.90)
HDL-C (mmol/L), median (P_25_, P_75_)	1.08 (1.01, 1.15)
Hypercholesterolemia, *n* (%)	1,001 (11.1)
Hypertriglyceridemia, *n* (%)	2021 (22.4)
Low HDL-C, *n* (%)	3,108 (34.4)
High LDL-C, *n* (%)	993 (11.0)

BMI, body mass index; TG, triglycerides; TC, total cholesterol; LDL-C, low-density lipoprotein cholesterol; HDL-C, high-density lipoprotein cholesterol; SD, standard deviation; P_25_, 25th percentile; P_75_, 75th percentile.

At baseline, the median concentrations of TG, TC, LDL-C, and HDL-C were 2.05, 5.63, 3.62, and 1.08 mmol/L, respectively. Among the four individual dyslipidemia components, low HDL-C was the most prevalent (34.4%), followed by hypertriglyceridemia (22.4%), hypercholesterolemia (11.1%), and high LDL-C (11.0%). The distributions of the 3-year average concentrations of ambient air pollutants are summarized in Table 2. Spearman correlation coefficients among the six pollutants are shown in Supplementary Table S4.

**Table 2:** Three-year average concentrations of air pollutants.

Pollutant	Summary statistics						
	Mean	SD	Minimum	P_25_	Median	P_75_	Maximum
PM_2.5_ (μg/m^3^)	52.00	5.29	35.60	48.23	52.47	57.23	62.03
PM_10_ (μg/m^3^)	103.46	14.16	70.03	92.63	102.33	111.73	130.60
O_3_ (μg/m^3^)	99.16	4.05	83.53	96.77	99.97	101.07	106.60
SO_2_ (μg/m^3^)	27.64	10.04	11.33	19.50	24.07	35.00	56.07
NO_2_ (μg/m^3^)	39.90	8.53	16.63	32.20	40.67	48.20	52.70
CO (mg/m^3^)	1.08	0.15	0.64	0.97	1.03	1.16	1.46

PM_2.5_, particulate matter with an aerodynamic diameter ≤ 2.5 μm; PM_10_, particulate matter with an aerodynamic diameter ≤ 10 μm; O_3_, ozone; SO_2_, sulfur dioxide; NO_2_, nitrogen dioxide; CO, carbon monoxide; SD, standard deviation; P25, 25th percentile; P75, 75th percentile.

### Associations between long-term air pollution exposure and lipid levels

Higher long-term exposure to PM_2.5_, PM_10_, O_3_, NO_2_, and SO_2_ was consistently associated with higher TG, TC, and LDL-C levels and lower HDL-C levels, with differing strengths across pollutants (Figure 1). Among the six pollutants, PM_2.5_ showed the strongest associations with all lipid outcomes. For each 10 μg/m^3^ increase in PM_2.5_, TG, TC, and LDL-C levels increased by 1.85, 1.70, and 1.81%, respectively, whereas HDL-C decreased by 1.55%. By comparison, CO was positively associated with TC and LDL-C but was not significantly associated with TG or HDL-C. Detailed numerical estimates are presented in Supplementary Table S6.

**Figure 1:**
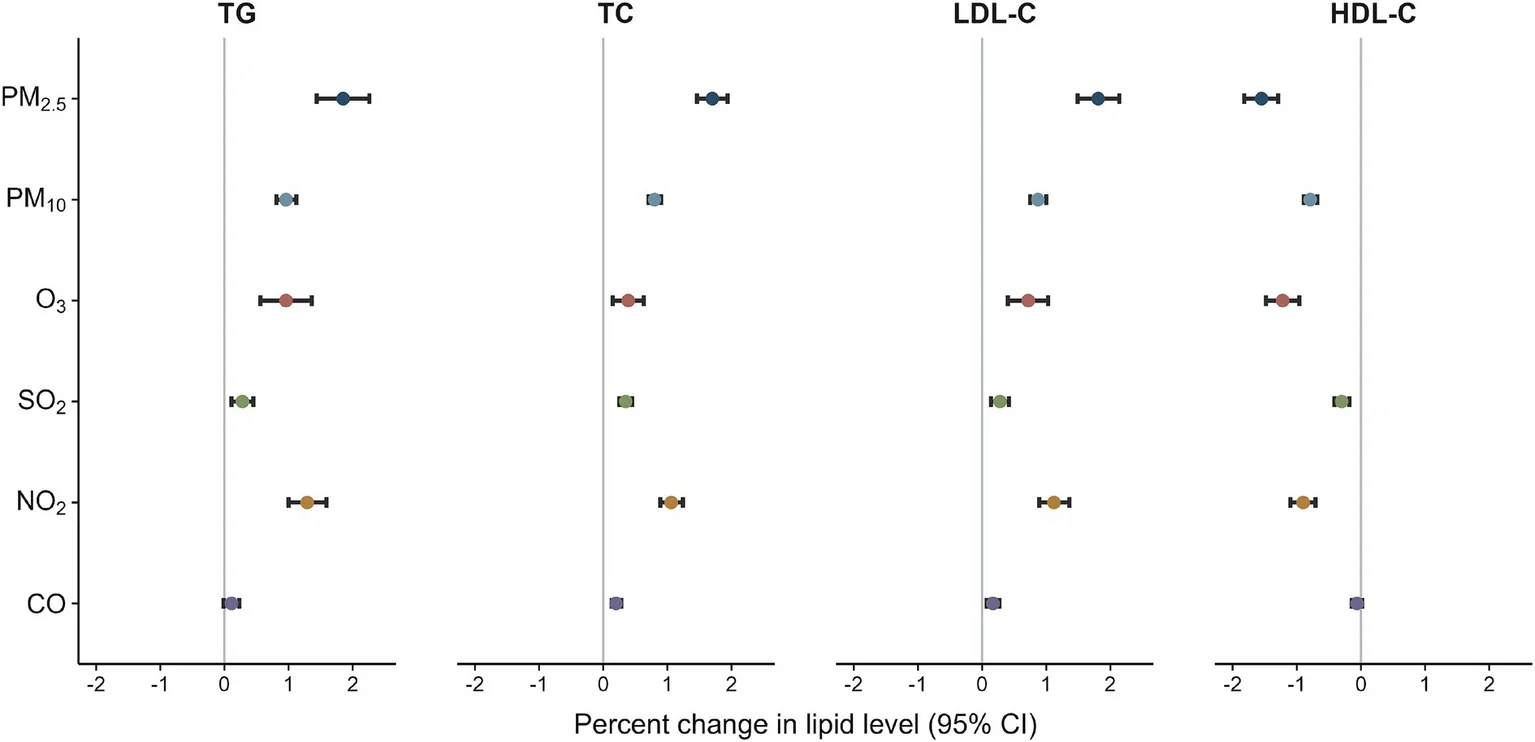
Forest plot of associations between long-term air pollution exposure and blood lipid levels. Effect estimates are expressed as percent changes with 95% confidence intervals per 10 μg/m^3^ increase in PM_2.5_, PM_10_, O_3_, SO_2_, and NO_2_ and per 0.1 mg/m^3^ increase in CO. Models were adjusted for age, sex, marital status, educational level, annual personal income, smoking status, alcohol consumption, physical activity, BMI, temperature, and relative humidity. PM_2.5_, particulate matter with an aerodynamic diameter ≤ 2.5 μm; PM_10_, particulate matter with an aerodynamic diameter ≤ 10 μm; O_3_, ozone; SO_2_, sulfur dioxide; NO_2_, nitrogen dioxide; CO, carbon monoxide; TG, triglycerides; TC, total cholesterol; LDL-C, low-density lipoprotein cholesterol; HDL-C, high-density lipoprotein cholesterol.

### Associations between long-term air pollution exposure and dyslipidemia

Higher long-term exposure to PM_2.5_, PM_10_, O_3_, NO_2_, and SO_2_ was associated with increased odds of hypertriglyceridemia, hypercholesterolemia, high LDL-C, and low HDL-C (Figure 2). Among the evaluated pollutants, PM_2.5_ generally showed the strongest associations with dyslipidemia outcomes, including hypertriglyceridemia (OR = 1.41), hypercholesterolemia (OR = 1.66), and low HDL-C (OR = 1.80), whereas O_3_ showed the strongest association with high LDL-C (OR = 1.52). By comparison, CO was significantly associated only with low HDL-C and showed no significant associations with the other dyslipidemia outcomes. Detailed numerical estimates are presented in Supplementary Table S7. In the secondary incident-outcome analyses, the directions of the associations were generally consistent with those observed in the primary repeated-measures analyses (Supplementary Table S23).

**Figure 2:**
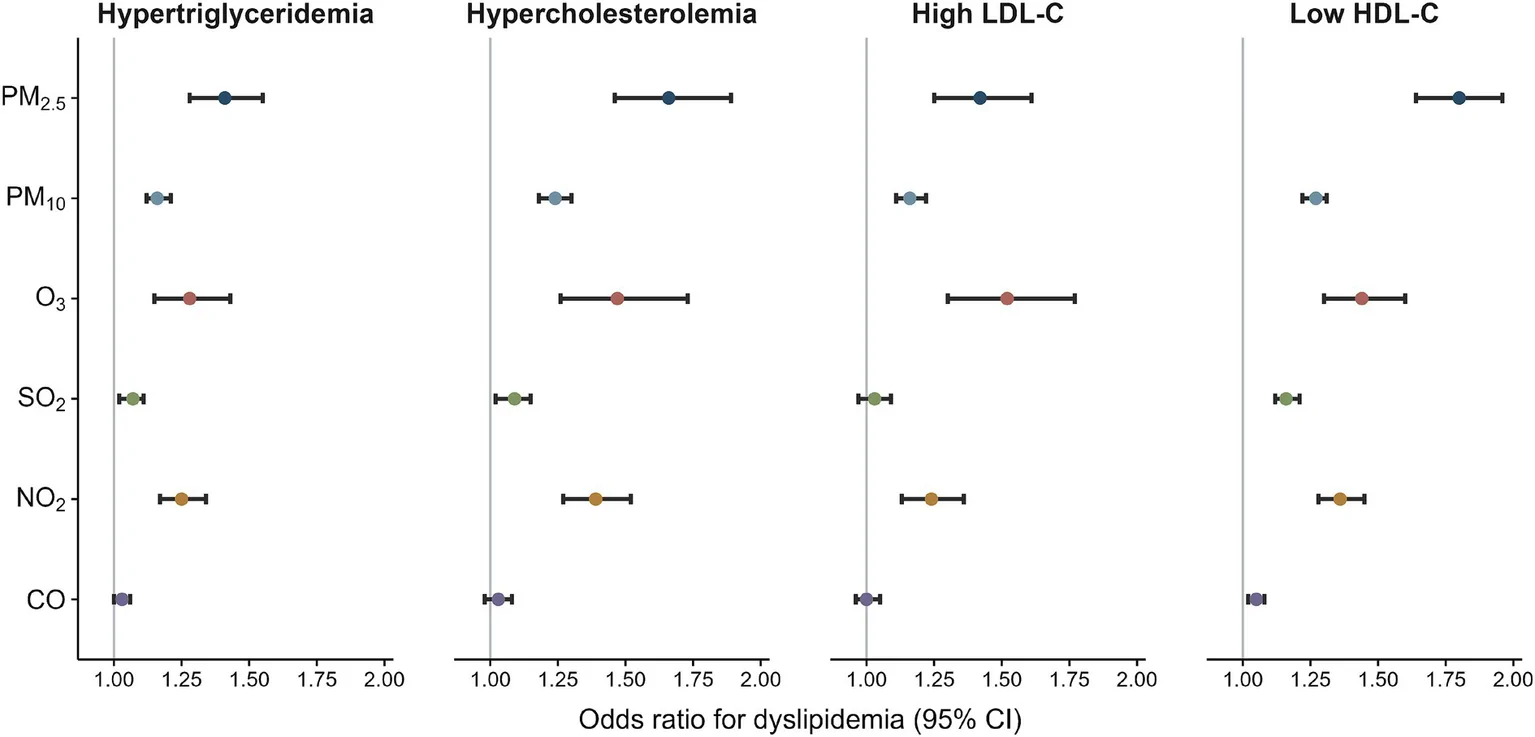
Forest plot of associations between long-term air pollution exposure and dyslipidemia. Effect estimates are expressed as odds ratios with 95% confidence intervals per 10 μg/m^3^ increase in PM_2.5_, PM_10_, O_3_, SO_2_, and NO_2_ and per 0.1 mg/m^3^ increase in CO. Models were adjusted for age, sex, marital status, educational level, annual personal income, smoking status, alcohol consumption, physical activity, BMI, temperature, and relative humidity. PM_2.5_, particulate matter with an aerodynamic diameter ≤ 2.5 μm; PM_10_, particulate matter with an aerodynamic diameter ≤ 10 μm; O_3_, ozone; SO_2_, sulfur dioxide; NO_2_, nitrogen dioxide; CO, carbon monoxide; TG, triglycerides; TC, total cholesterol; LDL-C, low-density lipoprotein cholesterol; HDL-C, high-density lipoprotein cholesterol.

### Stratified analyses

Stratified analyses suggested broadly similar directions of association across sex, BMI, and age subgroups, although the magnitude of the associations varied across several pollutant-lipid combinations (Figure 3A). Formal interaction tests showed that some subgroup differences were nominally significant, particularly for selected associations involving NO_2_, SO_2_, CO, and HDL-C. After FDR correction, the most robust interaction signals were mainly observed for age-stratified associations with HDL-C (Figure 3B). Detailed subgroup-specific estimates and interaction *p*-values are provided in Supplementary Tables S8, S9.

**Figure 3:**
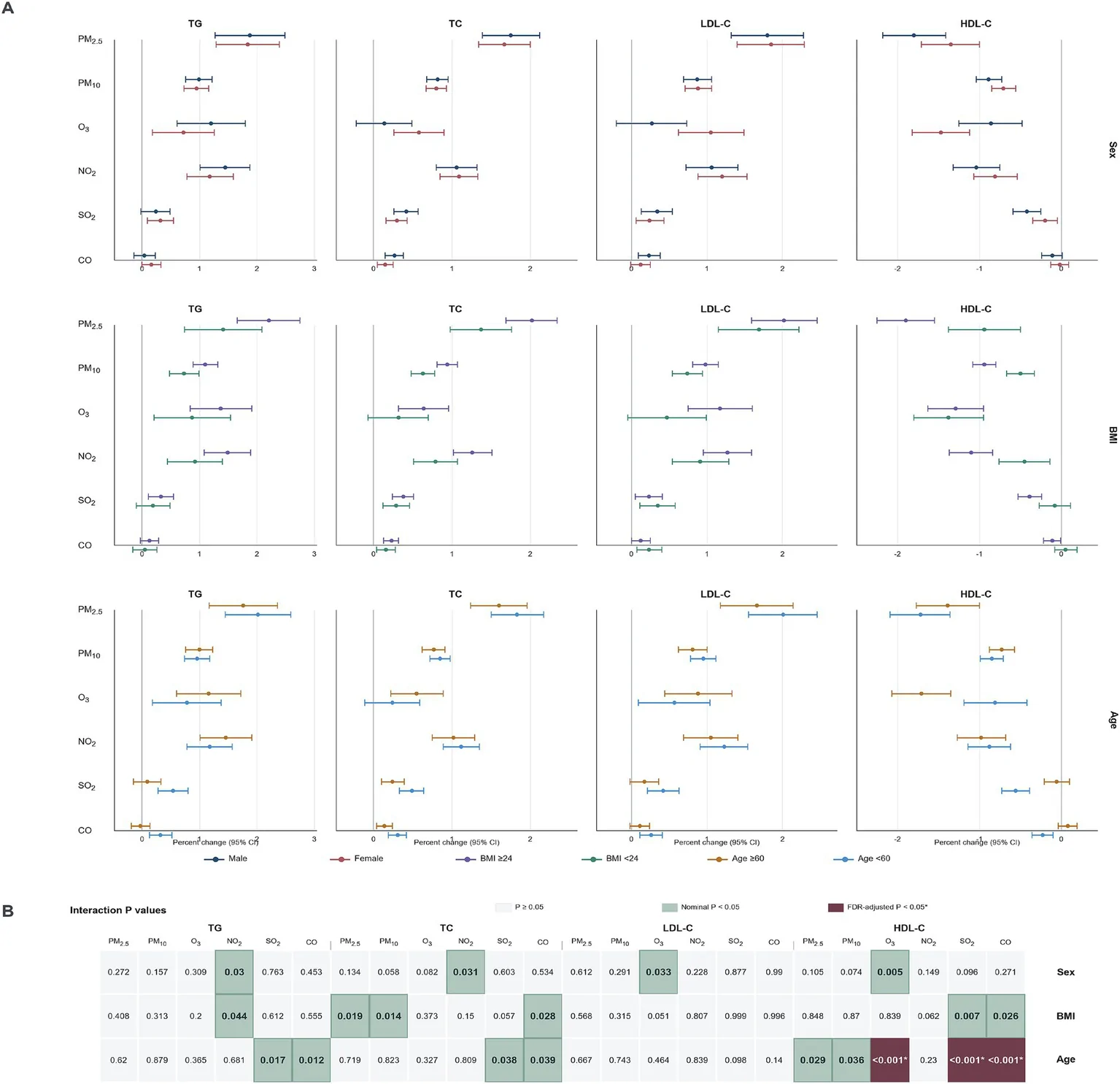
Stratified associations between long-term exposure to ambient air pollutants and blood lipid levels and corresponding interaction *p*-values. Panel **(A)** shows subgroup-specific percent changes and 95% confidence intervals from stratified linear mixed-effects models. Panel **(B)** displays the corresponding interaction *p*-values for all pollutant-lipid outcome combinations. Effect estimates are reported per 10 μg/m^3^ increase in PM_2.5_, PM_10_, O_3,_ SO_2_, and NO_2_, and per 0.1 mg/m^3^ increase in CO. Models were adjusted for age, sex, marital status, education, income, smoking, drinking, physical activity, BMI, temperature, and relative humidity, except for the corresponding stratification variable. Complete nominal and FDR-adjusted interaction *p*-values are provided in Supplementary Table S13. The asterisk indicates interactions that remained statistically significant after Benjamini-Hochberg false discovery rate correction. FDR, false discovery rate; PM_2.5_, particulate matter with an aerodynamic diameter ≤ 2.5 μm; PM_10_, particulate matter with an aerodynamic diameter ≤ 10 μm; O_3_, ozone; SO_2_, sulfur dioxide; NO_2_, nitrogen dioxide; CO, carbon monoxide; TG, triglycerides; TC, total cholesterol; LDL-C, low-density lipoprotein cholesterol; HDL-C, high-density lipoprotein cholesterol; BMI, body mass index; CI, confidence interval.

### Non-linear exposure–response relationships

Restricted cubic spline analyses suggested nonlinear exposure-response relationships between long-term PM_2.5_ exposure and TG, TC, LDL-C, and HDL-C levels (all *P* for overall association <0.001 and all *P* for non-linearity <0.001; Figure 4 and Supplementary Table S15). The exposure–response curves showed nonlinear patterns across the observed exposure range, with TG, TC, and LDL-C showing overall increasing trends and HDL-C showing an overall decreasing trend as PM_2.5_ concentrations increased.

**Figure 4:**
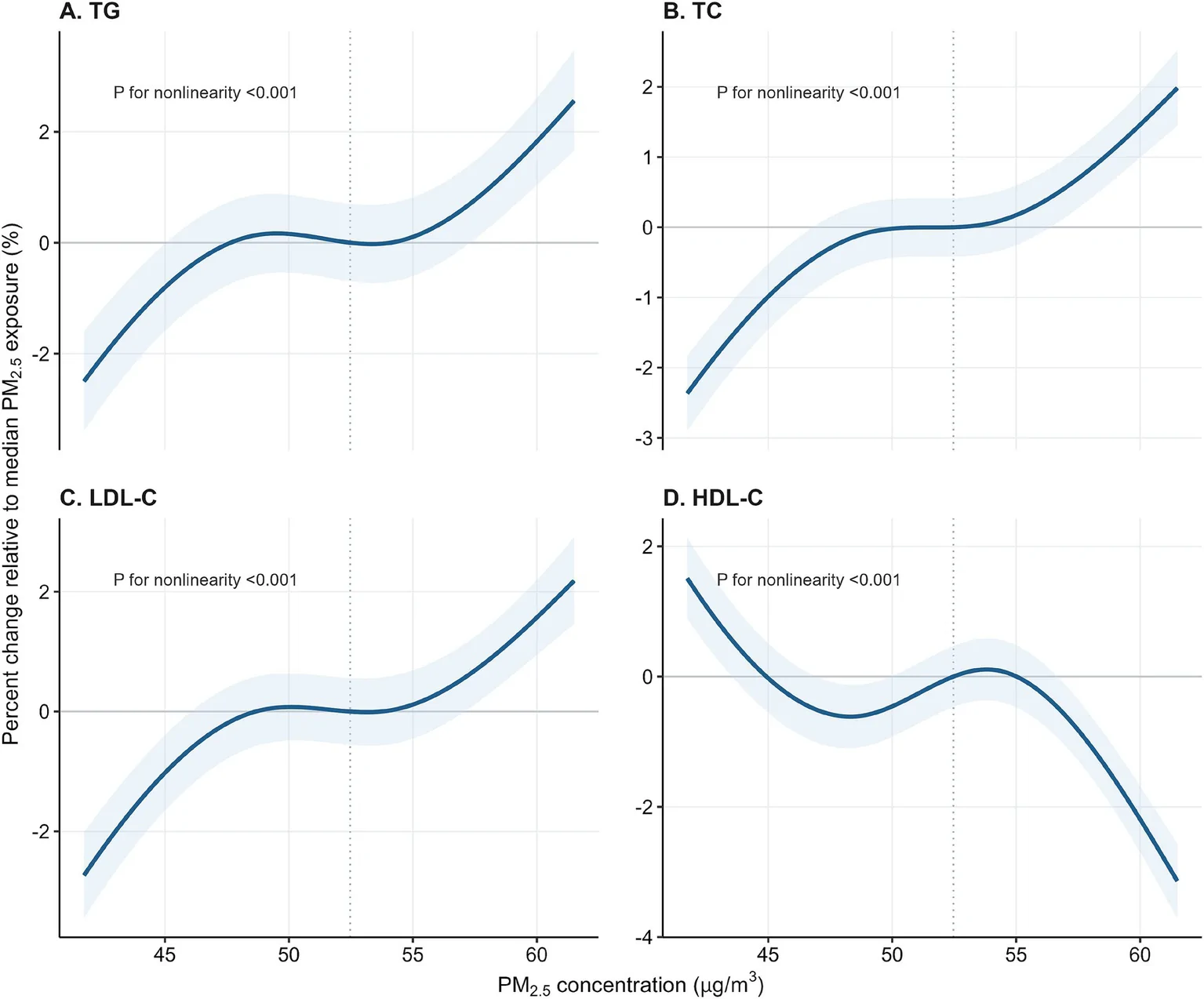
Restricted cubic spline analysis of the associations between long-term PM_2.5_ exposure and lipid levels. Panels **(A–D)** show the associations for TG, TC, LDL-C, and HDL-C, respectively. The curves show percent changes relative to the median PM_2.5_ concentration, and the shaded areas represent 95% confidence intervals. Models were adjusted for age, sex, marital status, education, income, smoking, drinking, physical activity, BMI, temperature, and relative humidity. Detailed spline parameters, including knot locations, reference values, and *p*-values for overall association and non-linearity, are presented in Supplementary Table S15. PM_2.5_, particulate matter with an aerodynamic diameter ≤ 2.5 μm; TG, triglycerides; TC, total cholesterol; LDL-C, low-density lipoprotein cholesterol; HDL-C, high-density lipoprotein cholesterol; CI, confidence interval.

### Multi-pollutant mixed-exposure analysis

The quantile g-computation-style mixed-effects analysis showed that joint exposure to multiple air pollutants was associated with higher TG, TC, and LDL-C levels and lower HDL-C levels (Figure 5A). Across all lipid outcomes, PM_2.5_ and O_3_ generally showed the largest weights in the overall mixed-exposure association, whereas PM_10_, NO_2_, SO_2_, and CO showed relatively smaller weights (Figure 5B).

**Figure 5:**
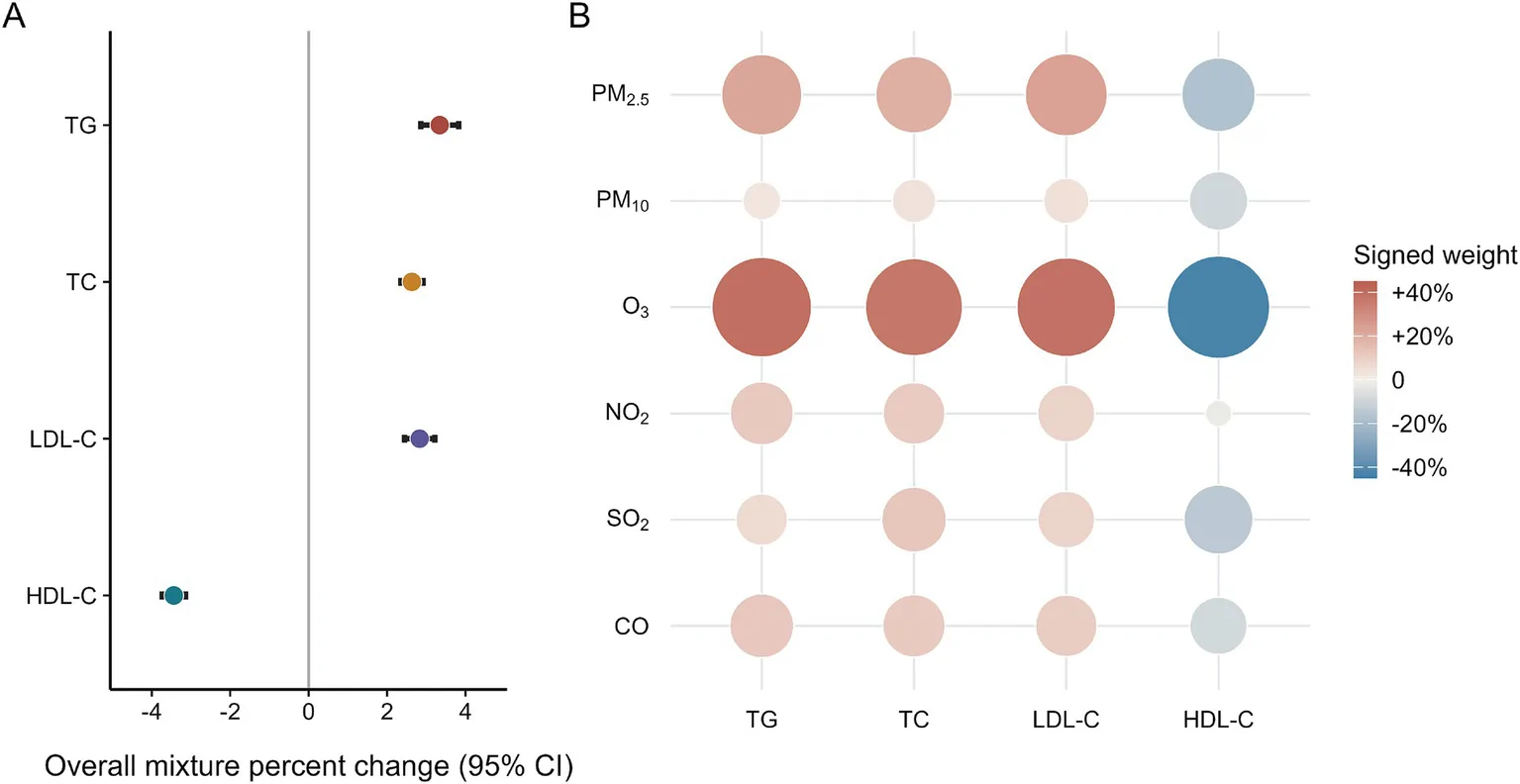
Quantile g-computation-style mixed-effects analysis of long-term exposure to multiple air pollutants and lipid levels. Panel **(A)** presents the overall mixture percent changes and 95% confidence intervals associated with a simultaneous one-quartile increase in the pollutant mixture. Panel **(B)** presents signed pollutant weights for each lipid outcome. The size of each bubble indicates the absolute value of the weight, and the color indicates the direction and magnitude of the signed weight. PM_2.5_, particulate matter with an aerodynamic diameter ≤ 2.5 μm; PM_10_, particulate matter with an aerodynamic diameter ≤ 10 μm; O_3_, ozone; NO_2_, nitrogen dioxide; SO_2_, sulfur dioxide; CO, carbon monoxide; TG, triglycerides; TC, total cholesterol; LDL-C, low-density lipoprotein cholesterol; HDL-C, high-density lipoprotein cholesterol; CI, confidence interval.

### Sensitivity analyses

Sensitivity analyses supported the robustness of the main findings. Results based on alternative exposure windows using 1-year and 5-year average pollutant concentrations were generally consistent with the primary analyses (Supplementary Table S19). The observed associations were also not materially changed after additional adjustment for histories of hypertension, diabetes, and coronary heart disease. Further adjustment for calendar year, season of examination, and prefecture-level region yielded broadly similar association patterns for most pollutant-lipid combinations, although estimates for several gaseous pollutants were more sensitive to temporal and regional adjustment (Supplementary Table S20). Exploratory leave-one-region-out analyses did not suggest that the main findings were driven entirely by a single prefecture-level region (Supplementary Table S21). When dyslipidemia outcomes were redefined using the 2023 Chinese lipid-management cutoffs, the directions of the associations were generally consistent with the primary analyses based on the 2007 guideline thresholds (Supplementary Table S22).

## Discussion

In this repeated-measures study of adults living in a heavily industrialized region of northern China, we found that long-term exposure to multiple ambient air pollutants was associated with adverse changes in lipid levels and higher odds of dyslipidemia. Among the pollutants examined, PM_2.5_ exhibited the strongest overall associations with adverse lipid outcomes. Notably, O_3_ was associated with greater reductions in HDL-C levels, higher odds of high LDL-C, and relatively large weights in the mixed-exposure analysis. These findings suggest that long-term exposure to ambient air pollution may be related not only to traditional cardiovascular events but also to earlier metabolic disturbances in lipid regulation. Furthermore, the observed non-linear exposure-response relationships and mixed-exposure associations highlight the complexity of the associations between long-term air pollution exposure and lipid metabolism in this heavily industrialized region.

The associations observed for PM_2.5_ in the present study are broadly consistent with previous epidemiological evidence linking long-term particulate matter exposure to adverse lipid metabolism. For example, findings from the China Multi-Ethnic Cohort demonstrated that long-term PM_2.5_ exposure was associated with higher TG and LDL-C levels and lower HDL-C levels (16), whereas another study in Chinese adults further reported higher odds of dyslipidemia associated with long-term PM_2.5_ exposure (17). Recent evidence from the nationwide ChinaHEART study also provides an important reference for interpreting our findings. This study reported that long-term exposure to PM_2.5_ and O_3_ was associated with less favorable lipid profiles, and that improvements in air quality after implementation of clean air policies were associated with reductions in several atherogenic lipid indicators (18). Similar associations have also been reported in prospective cohort studies conducted in Korea and Europe (19, 20), suggesting that adverse associations between PM_2.5_ and lipid metabolism may be observed across different populations and exposure settings. Nevertheless, the magnitude of these associations has varied among studies, which may be attributable to differences in exposure assessment methods, pollutant composition, population characteristics, ambient pollution levels, and statistical modeling strategies (7, 21). Building upon these previous findings, our study provides additional evidence from a heavily industrialized region in northern China by simultaneously evaluating continuous lipid levels and dyslipidemia outcomes using repeated health examination data.

Compared with particulate matter, epidemiological evidence regarding the metabolic associations of gaseous pollutants remains relatively limited and heterogeneous (22, 23). By comprehensively evaluating both lipid levels and clinically defined dyslipidemia, the present study provides additional epidemiological evidence supporting the potential role of O_3_ and NO_2_ in adverse lipid metabolism. Previous studies have likewise reported adverse associations of O_3_ exposure with lipid profiles, while studies conducted in both Chinese and European populations have linked NO_2_ exposure to unfavorable lipid profiles and dyslipidemia-related outcomes, lending further support to our findings (24–26). Importantly, our study was conducted in Shanxi Province, a major coal-producing and energy-intensive region where coal combustion, coking industries, industrial activities, and traffic emissions jointly contribute to a complex air pollution profile. This regional pollution profile may partly explain the relatively consistent associations observed between O_3_ and NO_2_ and multiple lipid outcomes in the present study (9–11).

Several biological mechanisms may account for the observed associations. A widely recognized pathway is that inhaled air pollutants can induce oxidative stress and systemic inflammation, leading to excessive production of reactive oxygen species (ROS) and pro-inflammatory cytokines (27–29). These responses may promote chronic low-grade inflammation, contribute to insulin resistance, and subsequently disrupt lipid homeostasis and promote metabolic dysfunction (30, 31). Furthermore, experimental studies suggest that air pollution can impair hepatic lipid metabolism by enhancing oxidative stress, promoting lipid accumulation, and altering the expression of key genes involved in lipid synthesis and oxidation (32, 33). Emerging evidence also indicates that O_3_ exposure may modulate hepatic lipid metabolism by activating key lipogenic regulators, including SREBP1, FASN, ACC1, and SCD1, while suppressing fatty acid oxidation, thereby contributing to hepatic lipid accumulation (34). Collectively, these interconnected pathways may help explain the observed associations between air pollution exposure and adverse lipid profiles.

The subgroup analyses provided further insight into potential differences in susceptibility to air pollution-related lipid changes. Although the directions of association were generally similar across subgroups, the estimated effects for several pollutant-lipid combinations tended to be stronger among participants with BMI ≥ 24 kg/m^2^. This pattern is consistent with previous epidemiological studies reporting greater susceptibility to air pollution-related metabolic disturbances among individuals with overweight or obesity (35–37). Excess adiposity is characterized by chronic low-grade inflammation, increased oxidative stress, and impaired insulin sensitivity, all of which may amplify the biological responses induced by air pollutants and increase vulnerability to lipid metabolic disturbances (38, 39). Nevertheless, the BMI-related interaction signals were attenuated after FDR correction, suggesting that these findings should be interpreted as suggestive rather than conclusive. Age-related differences were also observed, particularly for HDL-C. The more robust interaction signals after FDR correction were mainly found for age-stratified HDL-C associations, with the inverse association between O_3_ and HDL-C appearing stronger among participants aged ≥ 60 years. Aging is associated with altered lipid metabolism, reduced antioxidant capacity, vascular dysfunction, and greater cardiometabolic vulnerability, which may partly explain the stronger HDL-C response observed in older adults (40, 41). Taken together, these findings suggest that both excess adiposity and aging-related vulnerability may modify lipid responses to long-term air pollution exposure, although further studies are needed to confirm these subgroup-specific patterns.

The restricted cubic spline models suggested a potential nonlinear exposure-response relationship between long-term PM_2.5_ exposure and lipid parameters. These spline curves did not indicate a clear exposure threshold, but suggested that lipid responses to PM_2.5_ may vary across exposure levels. This variation may be related to exposure distribution, individual susceptibility, and biological responses at higher pollution levels. The biological processes related to PM_2.5_ exposure, including oxidative stress, systemic inflammation, endothelial dysfunction, lipid oxidation, and metabolic regulation, may show different response patterns across the exposure range rather than a simple linear pattern. At higher exposure levels, adaptive or saturation-like responses in inflammatory and metabolic pathways, changes in PM_2.5_ composition, and greater uncertainty in sparsely populated exposure ranges may also contribute to the observed nonlinear curve shapes (42–44). In real-world settings, individuals are usually exposed to multiple pollutants simultaneously, and single-pollutant models may not fully capture the overall health burden of ambient air pollution. Therefore, the quantile g-computation-style mixed-effects analysis provided a complementary assessment of the joint association between the pollutant mixture and lipid profiles. Because ambient air pollutants rarely occur in isolation, the pollutant-specific findings should be interpreted within the broader context of correlated exposures. The moderate-to-strong correlations among several primary pollutants in this study probably reflect shared emission sources and common spatial–temporal patterns in this industrialized region. Accordingly, single-pollutant estimates may represent not only the association of an individual pollutant but also that of a correlated pollution profile. The relatively larger weights of PM_2.5_ and O_3_ in the mixed-exposure analysis may partly reflect their roles within the correlated pollutant mixture, potentially related to systemic inflammation and metabolic disturbances induced by fine particulate matter, as well as the strong oxidative potential of O_3_ (29, 45, 46).

This study has several strengths. First, the repeated-measures design allowed us to account for within-participant correlations across multiple lipid examinations, providing more informative evidence than a purely cross-sectional analysis (16, 47, 48). Second, long-term air pollution exposure was assigned based on individual residential coordinates, which may reduce exposure misclassification compared with assigning exposure at the community or administrative-area level. Third, we conducted several supplementary analyses, including stratified analyses, alternative exposure windows, restricted cubic spline models, and quantile g-computation-style mixed-effects analysis, which provided additional insight into susceptible subgroups, potential non-linear exposure-response relationships, and the joint association between multipollutant exposure and lipid profiles.

Several limitations should be considered. First, although exposure was assigned using individual residential coordinates, information on time-activity patterns, workplace exposure, indoor air pollution, and residential mobility was unavailable, which may have introduced exposure measurement error. In addition, although we conducted supplementary analyses adjusting for calendar year, season of examination, and prefecture-level region, and further performed leave-one-region-out analyses, residual spatial confounding and local spatial clustering could not be fully excluded. More granular identifiers, such as community, county, screening center, or laboratory site, were not available in the de-identified dataset; therefore, these units could not be included as random effects or clustering units. Second, the study population was derived from a stroke-risk screening program rather than from a randomly selected sample of the general population. Participants included in this analysis may therefore differ from the broader adult population in baseline vascular risk, health awareness, healthcare access, and participation in repeated health examinations, and the exclusion of individuals with incomplete residential information, fewer than two valid lipid measurements, or missing key variables may have introduced selection bias. Third, despite adjustment for a broad range of covariates, residual confounding from unmeasured or incompletely measured factors cannot be excluded, particularly because examination-specific information on lipid-lowering medication use and detailed clinical treatment history was unavailable. Although histories of hypertension, coronary heart disease, and diabetes were additionally adjusted for in extended sensitivity models, these clinical history variables could not serve as reliable proxies for lipid-lowering medication use. Lipid-lowering medications may reduce TC, TG, and LDL-C levels and alter dyslipidemia classification, potentially leading to underestimation of dyslipidemia prevalence and attenuation of the observed associations.

## Conclusion

In conclusion, this repeated-measures study provides longitudinal evidence that long-term exposure to ambient air pollution is associated with adverse lipid profiles and may be related to higher odds of dyslipidemia among adults in Shanxi Province, China. The findings indicate that both particulate and gaseous pollutants were associated with unfavorable lipid profiles. Subgroup analyses further suggested potential heterogeneity in selected pollutant-lipid associations, although the most robust interaction signals after FDR correction were mainly observed for age-stratified HDL-C associations. By extending current evidence from a heavily industrialized region, this study adds evidence that ambient air pollution exposure is associated with lipid metabolism and underscores the need to consider long-term multipollutant exposure in cardiometabolic health research.

## Data Availability

The data analyzed in this study is subject to the following licenses/restrictions: the health-screening data analyzed in this study are not publicly available because of participant privacy and data-governance restrictions. De-identified data may be made available by the corresponding author upon reasonable request and with permission from the National Cerebrovascular Disease Big Data Platform. Requests to access these datasets should be directed to Changxin Li, lichangxin_123@sohu.com.

## Footnotes

1
https://www.chinasdc.cn/
