# Supplementary material for: Long-term exposure to ambient air pollution and lipid profiles: a longitudinal study in a heavily industrialized region of northern China
Source: Front Public Health. 2026 Oct 6;14:1950282. doi: 10.3389/fpubh.2026.1950282 (PMC13680454; doi:10.3389/fpubh.2026.1950282)
Supplement: Supplementary file 1 [file Data_Sheet_2_.docx]

Figure S1. Flowchart of participant selection.

The flowchart summarizes the initial screening population, sequential exclusion criteria, and final analytic sample included in the repeated-measures analyses.

Table S1. Missing data handling and model sample sizes.

| **Category** | **Item** | **n or model sample size** | **%** | **Handling** |
| --- | --- | --- | --- | --- |
| Initial sample | Initially eligible participants | 10,219 | 100.0 | Considered for eligibility |
| Sequential exclusion | Incomplete residential address information or residence duration <5 years | 348 | 3.4 | Excluded sequentially |
| Sequential exclusion | Fewer than two valid lipid measurements during follow-up | 516 | 5.0 | Excluded sequentially |
| Sequential exclusion | Missing key study variables | 315 | 3.1 | Excluded sequentially |
| Final analytic sample | Participants included in the main analysis | 9,040 | 88.5 | Included in analyses |
| Repeated observations | Lipid measurements included in the final analytic dataset | 19,113 observations | - | Included in repeated-measures models |
| Main continuous lipid models | LMMs for TG, TC, LDL-C, and HDL-C | 19,113 observations; 9,040 participants | - | Complete-case mixed-effects models |
| Main dyslipidemia models | GLMMs for hypertriglyceridemia, hypercholesterolemia, high LDL-C, and low HDL-C | 19,113 observations; 9,040 participants | - | Complete-case mixed-effects models |
| Sensitivity analyses | Alternative exposure windows and extended covariate models | 19,113 observations; 9,040 participants | - | Same analytic sample |

Abbreviations: LMM, linear mixed-effects model; GLMM, generalized linear mixed-effects model; TG, triglycerides; TC, total cholesterol; LDL-C, low-density lipoprotein cholesterol; HDL-C, high-density lipoprotein cholesterol. Percentages for exclusions and final participants were calculated using 10,219 initially eligible participants as the denominator. Exclusion criteria were applied sequentially to avoid double-counting participants who met more than one exclusion criterion. Model sample sizes refer to records with complete information on lipid measurements, exposure estimates, meteorological variables, and model covariates.

Table S2. Baseline characteristics of included and excluded participants.

| **Characteristic** | **Included (n=9,040)** | **Excluded (n=1,179)** | **SMD** | **P value** |
| --- | --- | --- | --- | --- |
| Age (years), mean ± SD | 58.57 ± 10.81 | 59.40 ± 10.74 | 0.077 | 0.013 |
| Female, n (%) | 4907 (54.3) | 719 (61.0) | 0.136 | <0.001 |
| Married, n (%) | 8387 (92.8) | 1117 (94.7) | 0.081 | 0.013 |
| Education level, n (%) |  |  |  |  |
| Primary school or below | 1690 (18.7) | 203 (17.2) |  | <0.001 |
| Junior/senior high school | 6244 (69.1) | 742 (62.9) |  |  |
| College or above | 1106 (12.2) | 234 (19.8) |  |  |
| Annual income >=20,000 CNY, n (%) | 4381 (48.5) | 547 (46.4) | 0.041 | 0.182 |
| Current smoking, n (%) | 1366 (15.1) | 177 (15.0) | 0.003 | 0.930 |
| Alcohol consumption, n (%) | 1445 (16.0) | 163 (13.8) | 0.061 | 0.055 |
| Physical inactivity, n (%) | 3379 (37.4) | 246 (20.9) | 0.370 | <0.001 |
| BMI (kg/m²), mean ± SD | 25.11 ± 3.44 | 25.08 ± 3.45 | 0.009 | 0.765 |
| Hypertension history, n (%) | 2211 (24.5) | 186 (15.8) | 0.218 | <0.001 |
| Diabetes history, n (%) | 689 (7.6) | 51 (4.3) | 0.139 | <0.001 |
| Heart disease history, n (%) | 271 (3.0) | 8 (0.7) | 0.173 | <0.001 |

Values are presented as mean ± SD or n (%). Included participants were those retained in the final analytic sample; excluded participants were those excluded because of incomplete residential information or residence duration less than five years, fewer than two valid lipid profile measurements, or missing key study variables. P values were derived from Welch's t test for continuous variables and chi-square tests for categorical variables. SMD values are presented as absolute standardized mean differences. BMI, body mass index; SMD, standardized mean difference.

Table S3. Characteristics of CHAP air pollution products used in this study.

| Pollutant | **CHAP product/version used** | **CHAP annual data used in this study** | **Spatial resolution** | **Temporal resolution** | **Exposure metric** | **Unit** | **Validation performance** |
| --- | --- | --- | --- | --- | --- | --- | --- |
| PM_2.5_ | ChinaHighPM_2.5_, V4 | 2014-2022 | 1 km × 1 km | Annual | Annual mean PM_2.5_ | μg/m³ | Daily CV-R² = 0.92; RMSE = 10.76 μg/m³ |
| PM_10_ | ChinaHighPM_10_, V4 | 2014-2022 | 1 km × 1 km | Annual | Annual mean PM_10_ | μg/m³ | Daily CV-R² = 0.90; RMSE = 21.12 μg/m³ |
| O_3_ | ChinaHighO_3_, V3 | 2014-2022 | 1 km × 1 km | Annual | Annual mean MDA8 O_3_ | μg/m³ | Daily CV-R² = 0.87; RMSE = 17.10 μg/m³ |
| NO_2_ | ChinaHighNO_2_, V1/V2 | 2014-2022 | 10 km × 10 km for 2014-2018; 1 km × 1 km for 2019-2022 | Annual | Annual mean NO_2_ | μg/m³ | Daily CV-R² = 0.93; RMSE = 4.89 μg/m³ for the 1-km product |
| SO_2_ | ChinaHighSO_2_, V1/V2 | 2014-2022 | 10 km × 10 km for 2014-2018; 1 km × 1 km for 2019-2022 | Annual | Annual mean SO_2_ | μg/m³ | Daily CV-R² = 0.84; RMSE ≈ 10.1–10.7 μg/m³ |
| CO | ChinaHighCO, V1/V2 | 2014-2022 | 10 km × 10 km for 2014-2018; 1 km × 1 km for 2019-2022 | Annual | Annual mean CO | mg/m³ | Daily CV-R² = 0.80; RMSE = 0.29 mg/m³ |

Abbreviations: CHAP, ChinaHighAirPollutants; PM_2.5_, particulate matter with aerodynamic diameter ≤ 2.5 μm; PM_10_, particulate matter with aerodynamic diameter ≤ 10 μm; O_3_, ozone; MDA8 O_3_, maximum daily 8-hour average ozone; NO_2_, nitrogen dioxide; SO_2_, sulfur dioxide; CO, carbon monoxide; CV-R2, cross-validation coefficient of determination; RMSE, root-mean-square error. Product version numbers are pollutant-specific and do not represent a unified versioning system across all pollutants. Because exposure windows were defined using complete calendar years before lipid examination, pollutant concentrations in 2023 were not used for exposure assignment.

Table S4. Spearman correlation coefficients among long-term air pollutant exposures.

| Pollutant | PM_2.5_ | PM_10_ | O_3_ | NO_2_ | SO_2_ | CO |
| --- | --- | --- | --- | --- | --- | --- |
| PM_2.5_ | 1.000 | 0.933 | -0.361 | 0.890 | 0.719 | 0.575 |
| PM_10_ | 0.933 | 1.000 | -0.278 | 0.975 | 0.557 | 0.384 |
| O_3_ | -0.361 | -0.278 | 1.000 | -0.206 | -0.677 | -0.659 |
| NO_2_ | 0.890 | 0.975 | -0.206 | 1.000 | 0.452 | 0.276 |
| SO_2_ | 0.719 | 0.557 | -0.677 | 0.452 | 1.000 | 0.945 |
| CO | 0.575 | 0.384 | -0.659 | 0.276 | 0.945 | 1.000 |

Values are Spearman correlation coefficients calculated using the long-term exposure variables included in the main analysis.

Table S5. Distribution of lipid parameters at the first eligible lipid examination.

| **Lipid parameter** | **Median (P25, P75)** | **Minimum** | **Maximum** |
| --- | --- | --- | --- |
| TG | 2.05 (1.87, 2.23) | 0.35 | 9.80 |
| TC | 5.63 (5.34, 5.95) | 3.99 | 8.94 |
| LDL-C | 3.62 (3.37, 3.90) | 2.38 | 5.62 |
| HDL-C | 1.08 (1.01, 1.15) | 0.69 | 1.97 |

Abbreviations: TG, triglycerides; TC, total cholesterol; LDL-C, low-density lipoprotein cholesterol; HDL-C, high-density lipoprotein cholesterol; P25, 25th percentile; P75, 75th percentile. All lipid values are expressed in mmol/L.

Figure S2. Distributions of lipid parameters at the first eligible lipid examination.

Histograms and density curves are shown for TG, TC, LDL-C, and HDL-C using one first eligible lipid examination per participant (n = 9,040). Shaded bands indicate the interquartile range and vertical lines indicate the median.

Table S6. Detailed associations between air pollutant increments and blood lipid levels.

| Pollutant | Percent change (95% CI)^a^ | | | |
| --- | --- | --- | --- | --- |
|  | TG | TC | LDL-C | HDL-C |
| PM_2.5_ | 1.85 (1.44, 2.26)^b^ | 1.70 (1.46, 1.94)^b^ | 1.81 (1.49, 2.14)^b^ | -1.55 (-1.82, -1.29)^b^ |
| PM_10_ | 0.96 (0.81, 1.12)^b^ | 0.80 (0.71, 0.90)^b^ | 0.87 (0.75, 1.00)^b^ | -0.79 (-0.89, -0.68)^b^ |
| O_3_ | 0.96 (0.56, 1.36)^b^ | 0.39 (0.15, 0.63)^b^ | 0.72 (0.40, 1.03)^b^ | -1.22 (-1.48, -0.96)^b^ |
| SO_2_ | 0.28 (0.11, 0.45)^b^ | 0.35 (0.25, 0.45)^b^ | 0.28 (0.14, 0.42)^b^ | -0.30 (-0.41, -0.18)^b^ |
| NO_2_ | 1.29 (1.00, 1.59)^b^ | 1.06 (0.89, 1.24)^b^ | 1.12 (0.89, 1.36)^b^ | -0.90 (-1.10, -0.71)^b^ |
| CO | 0.11 (-0.01, 0.23) | 0.20 (0.13, 0.28)^b^ | 0.17 (0.07, 0.27)^b^ | -0.06 (-0.14, 0.02) |

Values are percent changes (95% CIs) in lipid levels. Effect estimates are reported per 10 μg/m³ increase in PM_2.5_, PM_10_, O_3_, SO_2_, and NO_2_, and per 0.1 mg/m³ increase in CO.

^a^ Adjusted for age, sex, marital status, education, income, smoking, drinking, physical activity, BMI, temperature, and relative humidity.

^b^ Statistically significant association (*P* < 0.05).

Table S7. Detailed associations between air pollutant increments and dyslipidemia.

| Pollutant | Odds ratio (95% CI)^a^ | | | |
| --- | --- | --- | --- | --- |
|  | Hypertriglyceridemia | Hypercholesterolemia | High LDL-C | Low HDL-C |
| PM_2.5_ | 1.41 (1.28, 1.55)^b^ | 1.66 (1.46, 1.89)^b^ | 1.42 (1.25, 1.61)^b^ | 1.80 (1.64, 1.96)^b^ |
| PM_10_ | 1.16 (1.12, 1.21)^b^ | 1.24 (1.18, 1.30)^b^ | 1.16 (1.11, 1.22)^b^ | 1.27 (1.22, 1.31)^b^ |
| O_3_ | 1.28 (1.15, 1.43)^b^ | 1.47 (1.26, 1.73)^b^ | 1.52 (1.30, 1.77)^b^ | 1.44 (1.30, 1.60)^b^ |
| SO_2_ | 1.07 (1.02, 1.11)^b^ | 1.09 (1.02, 1.15)^b^ | 1.03 (0.97, 1.09) | 1.16 (1.12, 1.21)^b^ |
| NO_2_ | 1.25 (1.17, 1.34)^b^ | 1.39 (1.27, 1.52)^b^ | 1.24 (1.13, 1.36)^b^ | 1.36 (1.28, 1.45)^b^ |
| CO | 1.03 (1.00, 1.06) | 1.03 (0.98, 1.08) | 1.00 (0.96, 1.05) | 1.05 (1.02, 1.08)^b^ |

Values are odds ratios (95% CIs). Effect estimates are reported per 10 μg/m³ increase in PM_2.5_, PM_10_, O_3_, SO_2_, and NO_2_, and per 0.1 mg/m³ increase in CO.

^a^ Adjusted for age, sex, marital status, education, income, smoking, drinking, physical activity, BMI, temperature, and relative humidity.

^b^ Statistically significant association (*P* < 0.05).

Table S8. Stratified associations between long-term exposure to ambient air pollutants and lipid levels.

| Pollutant | Subgroup | TG Percent change (95% CI)^a^ | TC Percent change (95% CI)^a^ | LDL-C Percent change (95% CI)^a^ | HDL-C Percent change (95% CI)^a^ |
| --- | --- | --- | --- | --- | --- |
| PM_2.5_ | Sex |  |  |  |  |
|  | Male | 1.88 (1.27, 2.49)^b^ | 1.75 (1.39, 2.12)^b^ | 1.80 (1.32, 2.28)^b^ | -1.80 (-2.18, -1.41)^b^ |
|  | Female | 1.84 (1.29, 2.39)^b^ | 1.67 (1.34, 2.00)^b^ | 1.85 (1.40, 2.29)^b^ | -1.35 (-1.71, -1.00)^b^ |
|  | BMI |  |  |  |  |
|  | ≥24 | 2.21 (1.66, 2.75)^b^ | 2.02 (1.69, 2.34)^b^ | 2.02 (1.59, 2.46)^b^ | -1.90 (-2.25, -1.55)^b^ |
|  | <24 | 1.41 (0.74, 2.09)^b^ | 1.37 (0.98, 1.76)^b^ | 1.69 (1.15, 2.22)^b^ | -0.94 (-1.38, -0.50)^b^ |
|  | Age |  |  |  |  |
|  | ≥60 | 1.76 (1.17, 2.36)^b^ | 1.60 (1.24, 1.96)^b^ | 1.66 (1.18, 2.14)^b^ | -1.39 (-1.77, -1.00)^b^ |
|  | <60 | 2.02 (1.45, 2.59)^b^ | 1.83 (1.50, 2.17)^b^ | 2.01 (1.55, 2.46)^b^ | -1.72 (-2.09, -1.36)^b^ |
| PM_10_ | Sex |  |  |  |  |
|  | Male | 0.99 (0.76, 1.22)^b^ | 0.82 (0.68, 0.95)^b^ | 0.87 (0.69, 1.06)^b^ | -0.89 (-1.04, -0.73)^b^ |
|  | Female | 0.95 (0.73, 1.16)^b^ | 0.80 (0.67, 0.93)^b^ | 0.88 (0.71, 1.06)^b^ | -0.71 (-0.85, -0.56)^b^ |
|  | BMI |  |  |  |  |
|  | ≥24 | 1.10 (0.89, 1.32)^b^ | 0.94 (0.81, 1.07)^b^ | 0.98 (0.81, 1.15)^b^ | -0.94 (-1.08, -0.80)^b^ |
|  | <24 | 0.73 (0.48, 0.99)^b^ | 0.63 (0.48, 0.78)^b^ | 0.74 (0.54, 0.94)^b^ | -0.50 (-0.67, -0.33)^b^ |
|  | Age |  |  |  |  |
|  | ≥60 | 1.00 (0.76, 1.23)^b^ | 0.77 (0.62, 0.91)^b^ | 0.81 (0.62, 1.00)^b^ | -0.73 (-0.88, -0.57)^b^ |
|  | <60 | 0.96 (0.74, 1.18)^b^ | 0.85 (0.72, 0.98)^b^ | 0.95 (0.78, 1.12)^b^ | -0.85 (-0.99, -0.71)^b^ |
| O_3_ | Sex |  |  |  |  |
|  | Male | 1.20 (0.61, 1.80)^b^ | 0.14 (-0.22, 0.49) | 0.27 (-0.20, 0.73) | -0.86 (-1.25, -0.48)^b^ |
|  | Female | 0.72 (0.18, 1.26)^b^ | 0.58 (0.26, 0.90)^b^ | 1.05 (0.62, 1.49)^b^ | -1.47 (-1.82, -1.12)^b^ |
|  | BMI |  |  |  |  |
|  | ≥24 | 1.37 (0.84, 1.91)^b^ | 0.64 (0.32, 0.96)^b^ | 1.17 (0.75, 1.60)^b^ | -1.29 (-1.63, -0.95)^b^ |
|  | <24 | 0.87 (0.21, 1.54)^b^ | 0.32 (-0.07, 0.70) | 0.47 (-0.05, 0.99) | -1.38 (-1.80, -0.95)^b^ |
|  | Age |  |  |  |  |
|  | ≥60 | 1.16 (0.60, 1.72)^b^ | 0.55 (0.22, 0.89)^b^ | 0.88 (0.44, 1.33)^b^ | -1.71 (-2.07, -1.35)^b^ |
|  | <60 | 0.78 (0.18, 1.38)^b^ | 0.24 (-0.11, 0.59) | 0.57 (0.09, 1.04)^b^ | -0.81 (-1.19, -0.42)^b^ |
| SO_2_ | Sex |  |  |  |  |
|  | Male | 0.24 (-0.02, 0.49) | 0.42 (0.26, 0.57)^b^ | 0.34 (0.13, 0.54)^b^ | -0.42 (-0.59, -0.25)^b^ |
|  | Female | 0.32 (0.09, 0.55)^b^ | 0.30 (0.16, 0.43)^b^ | 0.24 (0.06, 0.43)^b^ | -0.20 (-0.35, -0.05)^b^ |
|  | BMI |  |  |  |  |
|  | ≥24 | 0.33 (0.11, 0.55)^b^ | 0.38 (0.24, 0.51)^b^ | 0.23 (0.05, 0.41)^b^ | -0.39 (-0.53, -0.24)^b^ |
|  | <24 | 0.19 (-0.10, 0.49) | 0.29 (0.12, 0.46)^b^ | 0.35 (0.11, 0.58)^b^ | -0.08 (-0.27, 0.11) |
|  | Age |  |  |  |  |
|  | ≥60 | 0.09 (-0.15, 0.33) | 0.24 (0.10, 0.39)^b^ | 0.17 (-0.02, 0.36) | -0.06 (-0.21, 0.10) |
|  | <60 | 0.54 (0.28, 0.80)^b^ | 0.49 (0.33, 0.64)^b^ | 0.42 (0.21, 0.63)^b^ | -0.56 (-0.73, -0.39)^b^ |
| NO_2_ | Sex |  |  |  |  |
|  | Male | 1.45 (1.01, 1.88)^b^ | 1.06 (0.80, 1.32)^b^ | 1.06 (0.72, 1.41)^b^ | -1.04 (-1.32, -0.75)^b^ |
|  | Female | 1.18 (0.78, 1.59)^b^ | 1.09 (0.85, 1.33)^b^ | 1.20 (0.88, 1.53)^b^ | -0.81 (-1.07, -0.54)^b^ |
|  | BMI |  |  |  |  |
|  | ≥24 | 1.49 (1.08, 1.89)^b^ | 1.26 (1.02, 1.51)^b^ | 1.27 (0.95, 1.59)^b^ | -1.10 (-1.37, -0.84)^b^ |
|  | <24 | 0.92 (0.44, 1.40)^b^ | 0.79 (0.51, 1.07)^b^ | 0.91 (0.54, 1.29)^b^ | -0.45 (-0.76, -0.14)^b^ |
|  | Age |  |  |  |  |
|  | ≥60 | 1.46 (1.01, 1.91)^b^ | 1.02 (0.75, 1.29)^b^ | 1.05 (0.69, 1.41)^b^ | -0.98 (-1.27, -0.68)^b^ |
|  | <60 | 1.18 (0.78, 1.57)^b^ | 1.12 (0.89, 1.35)^b^ | 1.23 (0.91, 1.54)^b^ | -0.88 (-1.14, -0.62)^b^ |
| CO | Sex |  |  |  |  |
|  | Male | 0.04 (-0.14, 0.23) | 0.27 (0.15, 0.38)^b^ | 0.23 (0.09, 0.38)^b^ | -0.11 (-0.24, 0.01) |
|  | Female | 0.16 (-0.00, 0.33) | 0.15 (0.05, 0.25)^b^ | 0.12 (-0.01, 0.25) | -0.02 (-0.13, 0.09) |
|  | BMI |  |  |  |  |
|  | ≥24 | 0.13 (-0.03, 0.29) | 0.23 (0.13, 0.32)^b^ | 0.12 (-0.00, 0.25) | -0.11 (-0.22, -0.01)^b^ |
|  | <24 | 0.05 (-0.16, 0.26) | 0.16 (0.04, 0.28)^b^ | 0.23 (0.07, 0.40)^b^ | 0.05 (-0.08, 0.19) |
|  | Age |  |  |  |  |
|  | ≥60 | -0.03 (-0.19, 0.14) | 0.14 (0.04, 0.24)^b^ | 0.11 (-0.02, 0.24) | 0.08 (-0.04, 0.19) |
|  | <60 | 0.32 (0.13, 0.52)^b^ | 0.31 (0.19, 0.42)^b^ | 0.26 (0.11, 0.41)^b^ | -0.23 (-0.36, -0.10)^b^ |

Effect estimates are reported as percent changes (95% CIs) per 10 μg/m³ increase in PM_2.5_, PM_10_, O_3_, SO_2_, and NO_2_, and per 0.1 mg/m³ increase in CO.

^a^ Adjusted for age, sex, marital status, education, income, smoking, drinking, physical activity, BMI, temperature, and relative humidity.

^b^ Statistically significant association (*P* < 0.05).

Table S9. *P* values for interaction in stratified analyses.

| Pollutant | Effect modifier | TG | TC | LDL-C | HDL-C |
| --- | --- | --- | --- | --- | --- |
| PM_2.5_ | Sex | 0.272 | 0.134 | 0.612 | 0.105 |
|  | BMI | 0.408 | 0.019 | 0.568 | 0.848 |
|  | Age | 0.620 | 0.719 | 0.667 | 0.029 |
| PM_10_ | Sex | 0.157 | 0.058 | 0.291 | 0.074 |
|  | BMI | 0.313 | 0.014 | 0.315 | 0.870 |
|  | Age | 0.879 | 0.823 | 0.743 | 0.036 |
| O_3_ | Sex | 0.309 | 0.082 | 0.033 | 0.005 |
|  | BMI | 0.200 | 0.373 | 0.051 | 0.839 |
|  | Age | 0.365 | 0.327 | 0.464 | <0.001 |
| SO_2_ | Sex | 0.763 | 0.603 | 0.877 | 0.096 |
|  | BMI | 0.612 | 0.057 | 0.999 | 0.007 |
|  | Age | 0.017 | 0.038 | 0.098 | <0.001 |
| NO_2_ | Sex | 0.030 | 0.031 | 0.228 | 0.149 |
|  | BMI | 0.044 | 0.150 | 0.807 | 0.062 |
|  | Age | 0.681 | 0.809 | 0.839 | 0.230 |
| CO | Sex | 0.453 | 0.534 | 0.990 | 0.271 |
|  | BMI | 0.555 | 0.028 | 0.996 | 0.026 |
|  | Age | 0.012 | 0.039 | 0.140 | <0.001 |

Interaction *P* values were obtained by adding multiplicative interaction terms between pollutant concentrations and subgroup variables to the mixed-effects models.

Table S10. Baseline characteristics according to BMI category.

| **Characteristic** | **Overall** | **BMI <24 kg/m²** | **BMI ≥24 kg/m²** | **P value** | **SMD** |
| --- | --- | --- | --- | --- | --- |
| N | 9040 | 3503 | 5537 |  |  |
| Age, years, mean ± SD | 58.57 ± 10.81 | 58.82 ± 11.14 | 58.41 ± 10.59 | 0.083 | 0.038 |
| BMI, kg/m², mean ± SD | 25.09 ± 3.39 | 21.99 ± 1.53 | 27.05 ± 2.71 | <0.001 | 2.178 |
| Sex, n (%) |  |  |  | 0.008 | 0.058 |
| Female | 4907 (54.3) | 1963 (56.0) | 2944 (53.2) |  |  |
| Male | 4133 (45.7) | 1540 (44.0) | 2593 (46.8) |  |  |
| Marital status, n (%) |  |  |  | 0.485 | 0.016 |
| Married | 8402 (92.9) | 3247 (92.7) | 5155 (93.1) |  |  |
| Widowed/Single/Divorced | 638 (7.1) | 256 (7.3) | 382 (6.9) |  |  |
| Education level, n (%) |  |  |  | 0.357 | 0.031 |
| Primary school or below | 1678 (18.6) | 643 (18.4) | 1035 (18.7) |  |  |
| Junior or senior high school | 6244 (69.1) | 2405 (68.7) | 3839 (69.3) |  |  |
| College or above | 1118 (12.4) | 455 (13.0) | 663 (12.0) |  |  |
| Annual personal income, n (%) |  |  |  | <0.001 | 0.102 |
| <5,000 RMB/year | 2064 (22.8) | 745 (21.3) | 1319 (23.8) |  |  |
| 5,000-9,999 RMB/year | 1396 (15.4) | 477 (13.6) | 919 (16.6) |  |  |
| 10,000-19,999 RMB/year | 1199 (13.3) | 474 (13.5) | 725 (13.1) |  |  |
| ≥20,000 RMB/year | 4381 (48.5) | 1807 (51.6) | 2574 (46.5) |  |  |
| Smoking status, n (%) |  |  |  | 0.033 | 0.047 |
| No | 7633 (84.4) | 2994 (85.5) | 4639 (83.8) |  |  |
| Yes | 1407 (15.6) | 509 (14.5) | 898 (16.2) |  |  |
| Alcohol consumption, n (%) |  |  |  | 0.070 | 0.040 |
| No | 7278 (80.5) | 2854 (81.5) | 4424 (79.9) |  |  |
| Yes | 1762 (19.5) | 649 (18.5) | 1113 (20.1) |  |  |
| Physical activity, n (%) |  |  |  | 0.171 | 0.030 |
| Regular activity | 4821 (53.3) | 1836 (52.4) | 2985 (53.9) |  |  |
| Physical inactivity | 4219 (46.7) | 1667 (47.6) | 2552 (46.1) |  |  |
| Hypertension history, n (%) |  |  |  | <0.001 | 0.261 |
| No | 6829 (75.5) | 2881 (82.2) | 3948 (71.3) |  |  |
| Yes | 2211 (24.5) | 622 (17.8) | 1589 (28.7) |  |  |
| Diabetes history, n (%) |  |  |  | <0.001 | 0.130 |
| No | 8351 (92.4) | 3308 (94.4) | 5043 (91.1) |  |  |
| Yes | 689 (7.6) | 195 (5.6) | 494 (8.9) |  |  |
| Heart disease history, n (%) |  |  |  | 0.002 | 0.070 |
| No | 8769 (97.0) | 3423 (97.7) | 5346 (96.6) |  |  |
| Yes | 271 (3.0) | 80 (2.3) | 191 (3.4) |  |  |

Values are presented for the first eligible lipid examination of each participant. *P* values were derived from Welch's t test for normally summarized continuous variables, Wilcoxon rank-sum tests for lipid variables summarized as medians, and chi-square tests for categorical variables. For categorical variables with more than two levels, the SMD represents the maximum absolute level-specific standardized mean difference. BMI, body mass index; SMD, standardized mean difference;

Table S11. Nominal and FDR-adjusted *P* values for main analyses of lipid levels.

| **Outcome** | **Pollutant** | **Increment** | **% change (95% CI)** | **Nominal P** | **FDR-adjusted P** |
| --- | --- | --- | --- | --- | --- |
| TG | PM_2.5_ | per 10 μg/m³ | 1.85 (1.44, 2.26) | <0.001 | <0.001 |
| TG | PM_10_ | per 10 μg/m³ | 0.96 (0.81, 1.12) | <0.001 | <0.001 |
| TG | O_3_ | per 10 μg/m³ | 0.96 (0.56, 1.36) | <0.001 | <0.001 |
| TG | NO_2_ | per 10 μg/m³ | 1.29 (1.00, 1.59) | <0.001 | <0.001 |
| TG | SO_2_ | per 10 μg/m³ | 0.28 (0.11, 0.45) | 0.001 | 0.001 |
| TG | CO | per 0.1 mg/m³ | 0.11 (-0.01, 0.23) | 0.083 | 0.087 |
| TC | PM_2.5_ | per 10 μg/m³ | 1.70 (1.46, 1.94) | <0.001 | <0.001 |
| TC | PM_10_ | per 10 μg/m³ | 0.80 (0.71, 0.90) | <0.001 | <0.001 |
| TC | O_3_ | per 10 μg/m³ | 0.39 (0.15, 0.63) | 0.001 | 0.001 |
| TC | NO_2_ | per 10 μg/m³ | 1.06 (0.89, 1.24) | <0.001 | <0.001 |
| TC | SO_2_ | per 10 μg/m³ | 0.35 (0.25, 0.45) | <0.001 | <0.001 |
| TC | CO | per 0.1 mg/m³ | 0.20 (0.13, 0.28) | <0.001 | <0.001 |
| LDL-C | PM_2.5_ | per 10 μg/m³ | 1.81 (1.49, 2.14) | <0.001 | <0.001 |
| LDL-C | PM_10_ | per 10 μg/m³ | 0.87 (0.75, 1.00) | <0.001 | <0.001 |
| LDL-C | O_3_ | per 10 μg/m³ | 0.72 (0.40, 1.03) | <0.001 | <0.001 |
| LDL-C | NO_2_ | per 10 μg/m³ | 1.12 (0.89, 1.36) | <0.001 | <0.001 |
| LDL-C | SO_2_ | per 10 μg/m³ | 0.28 (0.14, 0.42) | <0.001 | <0.001 |
| LDL-C | CO | per 0.1 mg/m³ | 0.17 (0.07, 0.27) | <0.001 | <0.001 |
| HDL-C | PM_2.5_ | per 10 μg/m³ | -1.55 (-1.82, -1.29) | <0.001 | <0.001 |
| HDL-C | PM_10_ | per 10 μg/m³ | -0.79 (-0.89, -0.68) | <0.001 | <0.001 |
| HDL-C | O_3_ | per 10 μg/m³ | -1.22 (-1.48, -0.96) | <0.001 | <0.001 |
| HDL-C | NO_2_ | per 10 μg/m³ | -0.90 (-1.10, -0.71) | <0.001 | <0.001 |
| HDL-C | SO_2_ | per 10 μg/m³ | -0.30 (-0.41, -0.18) | <0.001 | <0.001 |
| HDL-C | CO | per 0.1 mg/m³ | -0.06 (-0.14, 0.02) | 0.135 | 0.135 |

Abbreviations: FDR, false discovery rate; CI, confidence interval. FDR-adjusted *P* values were calculated using the Benjamini-Hochberg method.

Table S12. Nominal and FDR-adjusted *P* values for main analyses of dyslipidemia.

| **Outcome** | **Pollutant** | **Increment** | **OR (95% CI)** | **Nominal P** | **FDR-adjusted P** |
| --- | --- | --- | --- | --- | --- |
| Hypertriglyceridemia | PM_2.5_ | per 10 μg/m³ | 1.41 (1.28, 1.55) | <0.001 | <0.001 |
| Hypertriglyceridemia | PM_10_ | per 10 μg/m³ | 1.16 (1.12, 1.21) | <0.001 | <0.001 |
| Hypertriglyceridemia | O_3_ | per 10 μg/m³ | 1.28 (1.15, 1.43) | <0.001 | <0.001 |
| Hypertriglyceridemia | NO_2_ | per 10 μg/m³ | 1.25 (1.17, 1.34) | <0.001 | <0.001 |
| Hypertriglyceridemia | SO_2_ | per 10 μg/m³ | 1.07 (1.02, 1.11) | 0.004 | 0.005 |
| Hypertriglyceridemia | CO | per 0.1 mg/m³ | 1.03 (1.00, 1.06) | 0.081 | 0.093 |
| Hypercholesterolemia | PM_2.5_ | per 10 μg/m³ | 1.66 (1.46, 1.89) | <0.001 | <0.001 |
| Hypercholesterolemia | PM_10_ | per 10 μg/m³ | 1.24 (1.18, 1.30) | <0.001 | <0.001 |
| Hypercholesterolemia | O_3_ | per 10 μg/m³ | 1.47 (1.26, 1.73) | <0.001 | <0.001 |
| Hypercholesterolemia | NO_2_ | per 10 μg/m³ | 1.39 (1.27, 1.52) | <0.001 | <0.001 |
| Hypercholesterolemia | SO_2_ | per 10 μg/m³ | 1.09 (1.02, 1.15) | 0.006 | 0.007 |
| Hypercholesterolemia | CO | per 0.1 mg/m³ | 1.03 (0.98, 1.08) | 0.218 | 0.238 |
| High LDL-C | PM_2.5_ | per 10 μg/m³ | 1.42 (1.25, 1.61) | <0.001 | <0.001 |
| High LDL-C | PM_10_ | per 10 μg/m³ | 1.16 (1.11, 1.22) | <0.001 | <0.001 |
| High LDL-C | O_3_ | per 10 μg/m³ | 1.52 (1.30, 1.77) | <0.001 | <0.001 |
| High LDL-C | NO_2_ | per 10 μg/m³ | 1.24 (1.13, 1.36) | <0.001 | <0.001 |
| High LDL-C | SO_2_ | per 10 μg/m³ | 1.03 (0.97, 1.09) | 0.330 | 0.344 |
| High LDL-C | CO | per 0.1 mg/m³ | 1.00 (0.96, 1.05) | 0.871 | 0.871 |
| Low HDL-C | PM_2.5_ | per 10 μg/m³ | 1.80 (1.64, 1.96) | <0.001 | <0.001 |
| Low HDL-C | PM_10_ | per 10 μg/m³ | 1.27 (1.22, 1.31) | <0.001 | <0.001 |
| Low HDL-C | O_3_ | per 10 μg/m³ | 1.44 (1.30, 1.60) | <0.001 | <0.001 |
| Low HDL-C | NO_2_ | per 10 μg/m³ | 1.36 (1.28, 1.45) | <0.001 | <0.001 |
| Low HDL-C | SO_2_ | per 10 μg/m³ | 1.16 (1.12, 1.21) | <0.001 | <0.001 |
| Low HDL-C | CO | per 0.1 mg/m³ | 1.05 (1.02, 1.08) | 0.001 | 0.002 |

Abbreviations: FDR, false discovery rate; OR, odds ratio; CI, confidence interval. FDR-adjusted *P* values were calculated using the Benjamini-Hochberg method.

Table S13. Nominal and FDR-adjusted *P* values for interaction analyses.

| **Modifier** | **Outcome** | **Pollutant** | **Interaction P** | **FDR-adjusted P** |
| --- | --- | --- | --- | --- |
| Sex | TG | PM_2.5_ | 0.272 | 0.516 |
| Sex | TG | PM_10_ | 0.157 | 0.343 |
| Sex | TG | O_3_ | 0.309 | 0.556 |
| Sex | TG | NO_2_ | 0.030 | 0.169 |
| Sex | TG | SO_2_ | 0.763 | 0.916 |
| Sex | TG | CO | 0.453 | 0.694 |
| Sex | TC | PM_2.5_ | 0.134 | 0.331 |
| Sex | TC | PM_10_ | 0.058 | 0.191 |
| Sex | TC | O_3_ | 0.082 | 0.237 |
| Sex | TC | NO_2_ | 0.031 | 0.161 |
| Sex | TC | SO_2_ | 0.603 | 0.835 |
| Sex | TC | CO | 0.534 | 0.784 |
| Sex | LDL-C | PM_2.5_ | 0.612 | 0.816 |
| Sex | LDL-C | PM_10_ | 0.291 | 0.537 |
| Sex | LDL-C | O_3_ | 0.033 | 0.157 |
| Sex | LDL-C | NO_2_ | 0.228 | 0.470 |
| Sex | LDL-C | SO_2_ | 0.877 | 0.929 |
| Sex | LDL-C | CO | 0.990 | 1.000 |
| Sex | HDL-C | PM_2.5_ | 0.105 | 0.269 |
| Sex | HDL-C | PM_10_ | 0.074 | 0.222 |
| Sex | HDL-C | O_3_ | 0.005 | 0.086 |
| Sex | HDL-C | NO_2_ | 0.149 | 0.346 |
| Sex | HDL-C | SO_2_ | 0.096 | 0.264 |
| Sex | HDL-C | CO | 0.271 | 0.528 |
| BMI | TG | PM_2.5_ | 0.408 | 0.639 |
| BMI | TG | PM_10_ | 0.313 | 0.549 |
| BMI | TG | O_3_ | 0.200 | 0.423 |
| BMI | TG | NO_2_ | 0.044 | 0.168 |
| BMI | TG | SO_2_ | 0.612 | 0.831 |
| BMI | TG | CO | 0.555 | 0.799 |
| BMI | TC | PM_2.5_ | 0.019 | 0.151 |
| BMI | TC | PM_10_ | 0.014 | 0.141 |
| BMI | TC | O_3_ | 0.373 | 0.597 |
| BMI | TC | NO_2_ | 0.150 | 0.337 |
| BMI | TC | SO_2_ | 0.057 | 0.196 |
| BMI | TC | CO | 0.028 | 0.182 |
| BMI | LDL-C | PM_2.5_ | 0.568 | 0.801 |
| BMI | LDL-C | PM_10_ | 0.315 | 0.540 |
| BMI | LDL-C | O_3_ | 0.051 | 0.184 |
| BMI | LDL-C | NO_2_ | 0.807 | 0.953 |
| BMI | LDL-C | SO_2_ | 0.999 | 0.999 |
| BMI | LDL-C | CO | 0.996 | 1.000 |
| BMI | HDL-C | PM_2.5_ | 0.848 | 0.925 |
| BMI | HDL-C | PM_10_ | 0.870 | 0.935 |
| BMI | HDL-C | O_3_ | 0.839 | 0.943 |
| BMI | HDL-C | NO_2_ | 0.062 | 0.194 |
| BMI | HDL-C | SO_2_ | 0.007 | 0.104 |
| BMI | HDL-C | CO | 0.026 | 0.189 |
| Age | TG | PM_2.5_ | 0.620 | 0.812 |
| Age | TG | PM_10_ | 0.879 | 0.917 |
| Age | TG | O_3_ | 0.365 | 0.598 |
| Age | TG | NO_2_ | 0.681 | 0.860 |
| Age | TG | SO_2_ | 0.017 | 0.153 |
| Age | TG | CO | 0.012 | 0.138 |
| Age | TC | PM_2.5_ | 0.719 | 0.893 |
| Age | TC | PM_10_ | 0.823 | 0.940 |
| Age | TC | O_3_ | 0.327 | 0.548 |
| Age | TC | NO_2_ | 0.809 | 0.939 |
| Age | TC | SO_2_ | 0.038 | 0.161 |
| Age | TC | CO | 0.039 | 0.156 |
| Age | LDL-C | PM_2.5_ | 0.667 | 0.857 |
| Age | LDL-C | PM_10_ | 0.743 | 0.907 |
| Age | LDL-C | O_3_ | 0.464 | 0.696 |
| Age | LDL-C | NO_2_ | 0.839 | 0.930 |
| Age | LDL-C | SO_2_ | 0.098 | 0.262 |
| Age | LDL-C | CO | 0.140 | 0.336 |
| Age | HDL-C | PM_2.5_ | 0.029 | 0.176 |
| Age | HDL-C | PM_10_ | 0.036 | 0.161 |
| Age | HDL-C | O_3_ | <0.001 | 0.019 |
| Age | HDL-C | NO_2_ | 0.230 | 0.459 |
| Age | HDL-C | SO_2_ | <0.001 | <0.001 |
| Age | HDL-C | CO | <0.001 | 0.002 |

Abbreviations: FDR, false discovery rate. FDR-adjusted *P* values were calculated using the Benjamini-Hochberg method.

Table S14. Nominal and FDR-adjusted *P* values for restricted cubic spline analyses.

| **Outcome** | **Pollutant** | **P for nonlinearity** | **FDR-adjusted P** |
| --- | --- | --- | --- |
| TG | PM_2.5_ | <0.001 | <0.001 |
| TG | O_3_ | <0.001 | <0.001 |
| TC | PM_2.5_ | <0.001 | <0.001 |
| TC | O_3_ | <0.001 | <0.001 |
| LDL-C | PM_2.5_ | <0.001 | <0.001 |
| LDL-C | O_3_ | <0.001 | <0.001 |
| HDL-C | PM_2.5_ | <0.001 | <0.001 |
| HDL-C | O_3_ | <0.001 | <0.001 |

Abbreviations: FDR, false discovery rate. FDR-adjusted *P* values were calculated using the Benjamini-Hochberg method.

Table S15. Parameters and *P* values for restricted cubic spline analyses of PM_2.5_ and lipid levels.

| **Outcome** | **Spline df** | **Internal knots (μg/m³)** | **Boundary knots (μg/m³)** | **Reference value (μg/m³)** | **P for overall association** | **P for nonlinearity** |
| --- | --- | --- | --- | --- | --- | --- |
| TG | 3 | 48.80, 54.03 | 35.60, 62.03 | 52.47 | <0.001 | <0.001 |
| TC | 3 | 48.80, 54.03 | 35.60, 62.03 | 52.47 | <0.001 | <0.001 |
| LDL-C | 3 | 48.80, 54.03 | 35.60, 62.03 | 52.47 | <0.001 | <0.001 |
| HDL-C | 3 | 48.80, 54.03 | 35.60, 62.03 | 52.47 | <0.001 | <0.001 |

Restricted cubic spline analyses were performed using natural cubic spline terms with 3 degrees of freedom in linear mixed-effects models. The median PM_2.5_ concentration was used as the reference value. *P* values for overall association were obtained by comparing spline models with models without PM_2.5_, whereas *P* values for nonlinearity were obtained by comparing spline models with linear PM_2.5_ models.

Table S16. Quartile cut points used in the main quantile g-computation-style mixed-effects analysis.

| **Pollutant** | **Minimum** | **Q1** | **Median** | **Q3** | **Maximum** |
| --- | --- | --- | --- | --- | --- |
| PM_2.5_ | 35.60 | 48.23 | 52.47 | 57.23 | 62.03 |
| PM_10_ | 70.03 | 92.63 | 102.33 | 111.73 | 130.60 |
| O_3_ | 83.53 | 96.77 | 99.97 | 101.07 | 106.60 |
| NO_2_ | 16.63 | 32.20 | 40.67 | 48.20 | 52.70 |
| SO_2_ | 11.33 | 19.50 | 24.07 | 35.00 | 56.07 |
| CO | 0.64 | 0.97 | 1.03 | 1.16 | 1.46 |

Pollutants were categorized into quartiles and coded from 0 to 3 before simultaneous inclusion in the mixed-effects mixture model.

Table S17. Signed pollutant weights from the main quantile g-computation-style mixed-effects analysis.

| **Outcome** | **Pollutant** | **Coefficient** | **Signed weight (%)** | **Absolute weight (%)** | **Direction** |
| --- | --- | --- | --- | --- | --- |
| TG | PM_2.5_ | 0.008 | 23.6 | 23.6 | Positive |
| TG | PM_10_ | 0.001 | 3.2 | 3.2 | Positive |
| TG | O_3_ | 0.014 | 41.6 | 41.6 | Positive |
| TG | NO_2_ | 0.004 | 12.1 | 12.1 | Positive |
| TG | SO_2_ | 0.002 | 6.8 | 6.8 | Positive |
| TG | CO | 0.004 | 12.6 | 12.6 | Positive |
| TC | PM_2.5_ | 0.005 | 20.6 | 20.6 | Positive |
| TC | PM_10_ | 0.001 | 4.4 | 4.4 | Positive |
| TC | O_3_ | 0.010 | 38.9 | 38.9 | Positive |
| TC | NO_2_ | 0.003 | 11.4 | 11.4 | Positive |
| TC | SO_2_ | 0.003 | 13.0 | 13.0 | Positive |
| TC | CO | 0.003 | 11.7 | 11.7 | Positive |
| LDL-C | PM_2.5_ | 0.007 | 25.0 | 25.0 | Positive |
| LDL-C | PM_10_ | 0.001 | 4.8 | 4.8 | Positive |
| LDL-C | O_3_ | 0.011 | 40.6 | 40.6 | Positive |
| LDL-C | NO_2_ | 0.003 | 9.2 | 9.2 | Positive |
| LDL-C | SO_2_ | 0.003 | 9.0 | 9.0 | Positive |
| LDL-C | CO | 0.003 | 11.3 | 11.3 | Positive |
| HDL-C | PM_2.5_ | -0.006 | -18.5 | 18.5 | Negative |
| HDL-C | PM_10_ | -0.003 | -9.7 | 9.7 | Negative |
| HDL-C | O_3_ | -0.016 | -44.7 | 44.7 | Negative |
| HDL-C | NO_2_ | -0.001 | -2.2 | 2.2 | Negative |
| HDL-C | SO_2_ | -0.005 | -15.5 | 15.5 | Negative |
| HDL-C | CO | -0.003 | -9.3 | 9.3 | Negative |

Signed weights indicate the direction and relative weight of each pollutant within the fitted mixture model. Absolute weights sum to 100% within each lipid outcome.

Table S18. Sensitivity analyses of mixture models addressing overlap between PM_2.5_ and PM_10_.

| **Mixture model** | **TG % change (95% CI)** | **TC % change (95% CI)** | **LDL-C % change (95% CI)** | **HDL-C % change (95% CI)** |
| --- | --- | --- | --- | --- |
| Main mixture | 3.34 (2.86, 3.82) | 2.63 (2.35, 2.92) | 2.83 (2.45, 3.22) | -3.44 (-3.73, -3.14) |
| Excluding PM_10_ | 3.34 (2.86, 3.82) | 2.63 (2.34, 2.91) | 2.82 (2.44, 3.21) | -3.42 (-3.72, -3.12) |
| Replacing PM_10_ with PMcoarse | 3.77 (3.27, 4.27) | 2.90 (2.60, 3.19) | 3.16 (2.76, 3.55) | -3.81 (-4.11, -3.51) |

The main mixture included PM_2.5_, PM_10_, O_3_, NO_2_, SO_2_, and CO. Sensitivity analyses excluded PM_10_ or replaced PM_10_ with PMcoarse, calculated as PM_10_ minus PM_2.5_. Effect estimates represent the percent change in lipid levels associated with a simultaneous one-quartile increase in all pollutants included in the corresponding mixture model.

Table S19. Sensitivity analyses of the associations between long-term air pollution exposure and lipid levels.

| Pollutant | Lipid | 5-year average Percent change (95% CI)^a^ | 1-year average Percent change (95% CI)^a^ | 3-year average Percent change (95% CI)^a,c^ |
| --- | --- | --- | --- | --- |
| PM_2.5_ | TG | 2.16 (1.74, 2.58)^b^ | 2.21 (1.76, 2.66)^b^ | 1.84 (1.43, 2.25)^b^ |
|  | TC | 1.88 (1.63, 2.13)^b^ | 2.02 (1.75, 2.28)^b^ | 1.70 (1.45, 1.94)^b^ |
|  | LDL-C | 2.08 (1.75, 2.42)^b^ | 2.16 (1.80, 2.52)^b^ | 1.81 (1.48, 2.13)^b^ |
|  | HDL-C | -1.74 (-2.01, -1.47)^b^ | -1.61 (-1.90, -1.32)^b^ | -1.55 (-1.81, -1.29)^b^ |
| PM_10_ | TG | 1.25 (1.08, 1.43)^b^ | 1.26 (1.10, 1.43)^b^ | 0.96 (0.80, 1.12)^b^ |
|  | TC | 1.00 (0.89, 1.11)^b^ | 1.01 (0.91, 1.11)^b^ | 0.80 (0.71, 0.90)^b^ |
|  | LDL-C | 1.13 (0.99, 1.27)^b^ | 1.15 (1.02, 1.28)^b^ | 0.87 (0.75, 1.00)^b^ |
|  | HDL-C | -1.03 (-1.15, -0.92)^b^ | -1.16 (-1.27, -1.06)^b^ | -0.79 (-0.89, -0.68)^b^ |
| O_3_ | TG | 0.21 (-0.21, 0.64) | 2.84 (2.13, 3.56)^b^ | 0.93 (0.54, 1.34)^b^ |
|  | TC | -0.18 (-0.43, 0.07) | 1.81 (1.39, 2.23)^b^ | 0.38 (0.15, 0.62)^b^ |
|  | LDL-C | 0.05 (-0.29, 0.38) | 2.19 (1.62, 2.75)^b^ | 0.72 (0.40, 1.03)^b^ |
|  | HDL-C | -0.95 (-1.23, -0.67)^b^ | -3.12 (-3.56, -2.67)^b^ | -1.22 (-1.48, -0.96)^b^ |
| SO_2_ | TG | 0.73 (0.52, 0.95)^b^ | 1.69 (1.27, 2.12)^b^ | 0.28 (0.11, 0.45)^b^ |
|  | TC | 0.72 (0.60, 0.85)^b^ | 1.58 (1.33, 1.83)^b^ | 0.35 (0.24, 0.45)^b^ |
|  | LDL-C | 0.74 (0.57, 0.91)^b^ | 1.58 (1.25, 1.92)^b^ | 0.28 (0.14, 0.41)^b^ |
|  | HDL-C | -0.60 (-0.74, -0.45)^b^ | -1.63 (-1.90, -1.36)^b^ | -0.30 (-0.41, -0.18)^b^ |
| NO_2_ | TG | 1.65 (1.34, 1.96)^b^ | 1.85 (1.56, 2.14)^b^ | 1.29 (0.99, 1.59)^b^ |
|  | TC | 1.27 (1.08, 1.45)^b^ | 1.49 (1.31, 1.66)^b^ | 1.07 (0.89, 1.24)^b^ |
|  | LDL-C | 1.44 (1.19, 1.69)^b^ | 1.66 (1.43, 1.89)^b^ | 1.13 (0.89, 1.36)^b^ |
|  | HDL-C | -1.24 (-1.45, -1.04)^b^ | -1.42 (-1.61, -1.23)^b^ | -0.91 (-1.10, -0.71)^b^ |
| CO | TG | 0.19 (0.03, 0.35)^b^ | 0.29 (0.12, 0.45)^b^ | 0.11 (-0.01, 0.24) |
|  | TC | 0.29 (0.20, 0.39)^b^ | 0.37 (0.27, 0.47)^b^ | 0.21 (0.13, 0.28)^b^ |
|  | LDL-C | 0.29 (0.16, 0.41)^b^ | 0.35 (0.22, 0.48)^b^ | 0.17 (0.07, 0.27)^b^ |
|  | HDL-C | -0.05 (-0.16, 0.05) | -0.16 (-0.27, -0.05)^b^ | -0.06 (-0.14, 0.02) |

Values are percent changes (95% CIs) in lipid levels.

^a^ Adjusted for age, sex, marital status, education, income, smoking, drinking, physical activity, BMI, temperature, and relative humidity.

^b^ Statistically significant association (P < 0.05).

^c^ Additionally adjusted for hypertension, diabetes, and coronary heart disease history based on the primary model.

Table S20. Temporal and regional sensitivity analyses of air pollution and blood lipid levels.

| **Pollutant** | **Lipid** | **Main model** | **Year + season adjusted** | **Year + season + prefecture adjusted** |
| --- | --- | --- | --- | --- |
| PM_2.5_ | TG | 1.85 (1.44, 2.26)^b^ | 2.94 (2.45, 3.44)^b^ | 2.25 (1.57, 2.93)^b^ |
| PM_2.5_ | TC | 1.70 (1.46, 1.94)^b^ | 2.41 (2.12, 2.71)^b^ | 2.05 (1.65, 2.45)^b^ |
| PM_2.5_ | LDL-C | 1.81 (1.49, 2.14)^b^ | 2.78 (2.38, 3.17)^b^ | 2.22 (1.68, 2.76)^b^ |
| PM_2.5_ | HDL-C | -1.55 (-1.82, -1.29)^b^ | -2.52 (-2.83, -2.20)^b^ | -1.63 (-2.05, -1.21)^b^ |
| PM_10_ | TG | 0.96 (0.81, 1.12)^b^ | 1.05 (0.87, 1.23)^b^ | 1.13 (0.89, 1.37)^b^ |
| PM_10_ | TC | 0.80 (0.71, 0.90)^b^ | 0.84 (0.74, 0.95)^b^ | 0.92 (0.78, 1.07)^b^ |
| PM_10_ | LDL-C | 0.87 (0.75, 1.00)^b^ | 0.95 (0.80, 1.09)^b^ | 1.00 (0.80, 1.19)^b^ |
| PM_10_ | HDL-C | -0.79 (-0.89, -0.68)^b^ | -0.85 (-0.97, -0.73)^b^ | -0.89 (-1.04, -0.74)^b^ |
| O_3_ | TG | 0.96 (0.56, 1.36)^b^ | 5.29 (4.21, 6.38)^b^ | 2.27 (1.17, 3.39)^b^ |
| O_3_ | TC | 0.39 (0.15, 0.63)^b^ | 3.88 (3.25, 4.52)^b^ | 1.77 (1.12, 2.43)^b^ |
| O_3_ | LDL-C | 0.72 (0.40, 1.03)^b^ | 4.73 (3.88, 5.59)^b^ | 2.20 (1.32, 3.09)^b^ |
| O_3_ | HDL-C | -1.22 (-1.48, -0.96)^b^ | -5.44 (-6.07, -4.80)^b^ | -2.18 (-2.86, -1.49)^b^ |
| SO_2_ | TG | 0.28 (0.11, 0.45)^b^ | 2.66 (2.26, 3.06)^b^ | 0.71 (0.16, 1.27)^b^ |
| SO_2_ | TC | 0.35 (0.25, 0.45)^b^ | 2.10 (1.87, 2.34)^b^ | 0.81 (0.48, 1.14)^b^ |
| SO_2_ | LDL-C | 0.28 (0.14, 0.42)^b^ | 2.39 (2.07, 2.71)^b^ | 0.69 (0.25, 1.13)^b^ |
| SO_2_ | HDL-C | -0.30 (-0.41, -0.18)^b^ | -2.75 (-2.99, -2.50)^b^ | -0.74 (-1.09, -0.39)^b^ |
| NO_2_ | TG | 1.29 (1.00, 1.59)^b^ | 1.25 (0.95, 1.56)^b^ | 1.40 (0.97, 1.83)^b^ |
| NO_2_ | TC | 1.06 (0.89, 1.24)^b^ | 1.02 (0.84, 1.20)^b^ | 1.14 (0.89, 1.40)^b^ |
| NO_2_ | LDL-C | 1.12 (0.89, 1.36)^b^ | 1.10 (0.85, 1.34)^b^ | 1.12 (0.78, 1.46)^b^ |
| NO_2_ | HDL-C | -0.90 (-1.10, -0.71)^b^ | -0.85 (-1.05, -0.65)^b^ | -0.86 (-1.13, -0.59)^b^ |
| CO | TG | 0.11 (-0.01, 0.23) | 3.65 (3.23, 4.08)^b^ | 2.13 (1.62, 2.63)^b^ |
| CO | TC | 0.20 (0.13, 0.28)^b^ | 2.87 (2.63, 3.12)^b^ | 1.86 (1.56, 2.15)^b^ |
| CO | LDL-C | 0.17 (0.07, 0.27)^b^ | 3.47 (3.13, 3.80)^b^ | 2.25 (1.85, 2.65)^b^ |
| CO | HDL-C | -0.06 (-0.14, 0.02) | -3.54 (-3.79, -3.28)^b^ | -1.82 (-2.13, -1.51)^b^ |

Values are percent changes (95% CIs) in lipid levels. Sensitivity models additionally adjusted for calendar year and season, and further for prefecture-level region.

^b^ Statistically significant association (*P* < 0.05).

Table S21. Leave-one-region-out sensitivity analyses of air pollution and blood lipid levels.

| **Pollutant** | **Lipid** | **Range of % change** | **Direction consistent** | **Significant models** | **Minimum observations** | **Maximum observations** |
| --- | --- | --- | --- | --- | --- | --- |
| PM_2.5_ | TG | 0.92 to 3.64 | Yes | 3/3 | 6859 | 16057 |
| PM_2.5_ | TC | 0.93 to 2.76 | Yes | 3/3 | 6859 | 16057 |
| PM_2.5_ | LDL-C | 0.99 to 3.44 | Yes | 3/3 | 6859 | 16057 |
| PM_2.5_ | HDL-C | -4.06 to -0.03 | Yes | 2/3 | 6859 | 16057 |
| PM_10_ | TG | 0.45 to 1.61 | Yes | 3/3 | 6859 | 16057 |
| PM_10_ | TC | 0.38 to 1.15 | Yes | 3/3 | 6859 | 16057 |
| PM_10_ | LDL-C | 0.39 to 1.40 | Yes | 3/3 | 6859 | 16057 |
| PM_10_ | HDL-C | -1.81 to -0.13 | Yes | 3/3 | 6859 | 16057 |
| O_3_ | TG | 3.12 to 8.53 | Yes | 3/3 | 6859 | 16057 |
| O_3_ | TC | 2.35 to 6.28 | Yes | 3/3 | 6859 | 16057 |
| O_3_ | LDL-C | 3.02 to 7.40 | Yes | 3/3 | 6859 | 16057 |
| O_3_ | HDL-C | -10.38 to -3.18 | Yes | 3/3 | 6859 | 16057 |
| SO_2_ | TG | 0.37 to 3.27 | Yes | 2/3 | 6859 | 16057 |
| SO_2_ | TC | 0.54 to 2.53 | Yes | 3/3 | 6859 | 16057 |
| SO_2_ | LDL-C | 0.45 to 2.87 | Yes | 3/3 | 6859 | 16057 |
| SO_2_ | HDL-C | -3.57 to -0.09 | Yes | 2/3 | 6859 | 16057 |
| NO_2_ | TG | 0.35 to 1.26 | Yes | 2/3 | 6859 | 16057 |
| NO_2_ | TC | 0.31 to 1.02 | Yes | 2/3 | 6859 | 16057 |
| NO_2_ | LDL-C | 0.24 to 0.99 | Yes | 1/3 | 6859 | 16057 |
| NO_2_ | HDL-C | -1.73 to 0.27 | No | 3/3 | 6859 | 16057 |
| CO | TG | 1.93 to 3.67 | Yes | 3/3 | 6859 | 16057 |
| CO | TC | 1.21 to 2.91 | Yes | 3/3 | 6859 | 16057 |
| CO | LDL-C | 1.22 to 3.55 | Yes | 3/3 | 6859 | 16057 |
| CO | HDL-C | -3.97 to -1.69 | Yes | 3/3 | 6859 | 16057 |

Values are ranges of percent-change estimates across three leave-one-region-out analyses. Leave-one-region-out analyses were performed by sequentially excluding one prefecture-level region at a time and refitting the year- and season-adjusted mixed-effects models.

Table S22. Sensitivity analyses of air pollutant increments and dyslipidemia using the 2023 Chinese lipid-management cutoffs.

| **Pollutant** | **Hypertriglyceridemia** | **Hypercholesterolemia** | **High LDL-C** | **Low HDL-C** |
| --- | --- | --- | --- | --- |
| PM_2.5_ | 1.34 (1.19, 1.51)^b^ | 1.58 (1.43, 1.74)^b^ | 1.53 (1.40, 1.66)^b^ | 1.82 (1.64, 2.02)^b^ |
| PM_10_ | 1.18 (1.13, 1.24)^b^ | 1.23 (1.18, 1.28)^b^ | 1.21 (1.18, 1.26)^b^ | 1.27 (1.22, 1.32)^b^ |
| O_3_ | 1.14 (1.01, 1.28)^b^ | 1.04 (0.94, 1.15) | 1.08 (0.99, 1.17) | 1.57 (1.38, 1.79)^b^ |
| SO_2_ | 1.08 (1.02, 1.13)^b^ | 1.17 (1.12, 1.22)^b^ | 1.12 (1.08, 1.17)^b^ | 1.16 (1.10, 1.21)^b^ |
| NO_2_ | 1.23 (1.13, 1.35)^b^ | 1.28 (1.19, 1.37)^b^ | 1.28 (1.20, 1.36)^b^ | 1.39 (1.29, 1.50)^b^ |
| CO | 1.03 (0.99, 1.07) | 1.12 (1.08, 1.15)^b^ | 1.07 (1.04, 1.10)^b^ | 1.04 (1.00, 1.08)^b^ |

Values are odds ratios (95% CIs) from generalized linear mixed-effects models with participant-specific random intercepts. Dyslipidemia outcomes were redefined using the 2023 Chinese lipid-management cutoffs: TC ≥5.2 mmol/L, TG ≥1.7 mmol/L, LDL-C ≥3.4 mmol/L, and HDL-C <1.0 mmol/L.

^b^ Statistically significant association (*P* < 0.05).

**Figure S3.** Representative model diagnostic plots for linear mixed-effects models of PM_2.5_ and lipid parameters. Left panels show standardized residuals versus fitted values, and right panels show Q-Q plots of standardized residuals. Diagnostic plots are shown for representative PM_2.5_ models for TG, TC, LDL-C, and HDL-C continuous outcomes.

Table S23. Secondary analyses of long-term air pollution exposure and incident dyslipidemia outcomes.

| **Outcome** | **Pollutant** | **OR (95% CI)** | ***P* value** | **Baseline-normal participants** | **Incident cases** |
| --- | --- | --- | --- | --- | --- |
| Incident dyslipidemia | PM_2.5_ | 1.87 (1.54, 2.28) | <0.001 | 4471 | 1172 |
| Incident dyslipidemia | PM_10_ | 1.16 (1.09, 1.24) | <0.001 | 4471 | 1172 |
| Incident dyslipidemia | O_3_ | 1.45 (0.99, 2.12) | 0.056 | 4471 | 1172 |
| Incident dyslipidemia | NO_2_ | 1.36 (1.21, 1.54) | <0.001 | 4471 | 1172 |
| Incident dyslipidemia | SO_2_ | 1.68 (1.45, 1.96) | <0.001 | 4471 | 1172 |
| Incident dyslipidemia | CO | 1.72 (1.42, 2.08) | <0.001 | 4471 | 1172 |
| Incident hypertriglyceridemia | PM_2.5_ | 1.55 (1.29, 1.87) | <0.001 | 7019 | 947 |
| Incident hypertriglyceridemia | PM_10_ | 1.11 (1.04, 1.18) | 0.001 | 7019 | 947 |
| Incident hypertriglyceridemia | O_3_ | 1.45 (1.01, 2.08) | 0.043 | 7019 | 947 |
| Incident hypertriglyceridemia | NO_2_ | 1.25 (1.12, 1.40) | <0.001 | 7019 | 947 |
| Incident hypertriglyceridemia | SO_2_ | 1.45 (1.26, 1.66) | <0.001 | 7019 | 947 |
| Incident hypertriglyceridemia | CO | 1.55 (1.29, 1.86) | <0.001 | 7019 | 947 |
| Incident hypercholesterolemia | PM_2.5_ | 1.39 (1.10, 1.76) | 0.005 | 8039 | 557 |
| Incident hypercholesterolemia | PM_10_ | 1.07 (0.99, 1.16) | 0.073 | 8039 | 557 |
| Incident hypercholesterolemia | O_3_ | 1.47 (0.96, 2.24) | 0.073 | 8039 | 557 |
| Incident hypercholesterolemia | NO_2_ | 1.21 (1.05, 1.40) | 0.008 | 8039 | 557 |
| Incident hypercholesterolemia | SO_2_ | 1.33 (1.12, 1.58) | <0.001 | 8039 | 557 |
| Incident hypercholesterolemia | CO | 1.43 (1.15, 1.78) | 0.001 | 8039 | 557 |
| Incident high LDL-C | PM_2.5_ | 1.42 (1.13, 1.78) | 0.002 | 8047 | 587 |
| Incident high LDL-C | PM_10_ | 1.08 (1.00, 1.16) | 0.045 | 8047 | 587 |
| Incident high LDL-C | O_3_ | 1.33 (0.88, 2.00) | 0.172 | 8047 | 587 |
| Incident high LDL-C | NO_2_ | 1.20 (1.04, 1.37) | 0.012 | 8047 | 587 |
| Incident high LDL-C | SO_2_ | 1.35 (1.15, 1.60) | <0.001 | 8047 | 587 |
| Incident high LDL-C | CO | 1.35 (1.09, 1.66) | 0.005 | 8047 | 587 |
| Incident low HDL-C | PM_2.5_ | 2.23 (1.82, 2.72) | <0.001 | 5932 | 977 |
| Incident low HDL-C | PM_10_ | 1.21 (1.14, 1.29) | <0.001 | 5932 | 977 |
| Incident low HDL-C | O_3_ | 1.09 (0.76, 1.55) | 0.653 | 5932 | 977 |
| Incident low HDL-C | NO_2_ | 1.46 (1.30, 1.65) | <0.001 | 5932 | 977 |
| Incident low HDL-C | SO_2_ | 1.95 (1.68, 2.26) | <0.001 | 5932 | 977 |
| Incident low HDL-C | CO | 1.67 (1.38, 2.01) | <0.001 | 5932 | 977 |

Note: Incident outcomes were analyzed among participants without the corresponding dyslipidemia component at the first eligible lipid examination. For overall incident dyslipidemia, participants with any abnormal lipid parameter at baseline were excluded. Each participant contributed one record indicating whether the corresponding outcome first occurred during follow-up. Models used baseline long-term air pollution exposure and adjusted for age, sex, marital status, educational level, annual personal income, smoking status, alcohol consumption, physical activity, BMI, baseline lipid level, follow-up time, lipid examination year, season, temperature, and relative humidity. For overall incident dyslipidemia, baseline TG, TC, LDL-C, and HDL-C levels were included. Effect estimates are odds ratios (ORs) and 95% confidence intervals (CIs) per 10 μg/m³ increase in PM_2.5_, PM_10_, O_3_, NO_2_, and SO_2_ and per 0.1 mg/m³ increase in CO. LDL-C, low-density lipoprotein cholesterol; HDL-C, high-density lipoprotein cholesterol.
